# WHO estimates of the global, regional, and national burden of 42 foodborne infectious and chemical hazards, 2000–21: an updated data synthesis

**DOI:** 10.1016/j.langlo.2026.103994

**Published:** 2026-06-03

**Authors:** Robin J Lake, Brecht Devleesschauwer, Shannon E Majowicz, Lucy J Robertson, Lea Sletting Jakobsen, Antonio Agudo, Sara M Pires, Martyn D Kirk, Elaine Scallan Walter, Karen H Keddy, Carlotta di Bari, Louise Vaes, Arie H Havelaar, Charlee Roberts, Tesfaye Gobena, Mirjam E Kretzschmar, Gabriela F Nane, Sandra Hoffmann, Lapo Mughini-Gras, Banchob Sripa, Kunihiro Kubota, Luria Leslie Founou, Li Bai, Mohammed Al Huthiel, Tety Rachmawati, Teresa Estrada-Garcia, Ashok Kumar, Fadi Al Natour, Steven Jaffee, Spencer Henson, Luc Ingenbleek, Richard Kumapley, Elaine Borghi, Yuki Minato, WHO Foodborne Disease Burden Epidemiology Reference Group for 2021–25

**Affiliations:** a Institute of Public Health and Forensic Science, Christchurch, New Zealand; b Department of Epidemiology and Public Health, Sciensano, Brussels, Belgium; c Department of Translational Physiology, Infectiology and Public health, Ghent University, Merelbeke, Belgium; d School of Public Health Sciences, University of Waterloo, Waterloo, ON, Canada; e Parasitology, Department of Paraclinical Sciences, Faculty of Veterinary Medicine, Norwegian University of Life Sciences, Ås, Norway; f National Food Institute, Technical University of Denmark, Kongens Lyngby, Denmark; g Unit of Nutrition and Cancer, Cancer Epidemiology Research Program, Catalan Institute of Oncology-ICO, Barcelona, Spain; h Nutrition and Cancer Group, Bellvitge Biomedical Research Institute-IDIBELL, L’Hospitalet de Llobregat, Barcelona, Spain; i National Centre for Epidemiology and Population Health, The Australian National University, Canberra, ACT, Australia; j Colorado School of Public Health, University of Colorado Anschutz Medical Campus, Aurora, CO, USA; k Department of Veterinary Tropical Diseases, Faculty of Veterinary Science, University of Pretoria, Pretoria, South Africa; l Emerging Pathogens Institute, Global Food Systems Institute, Department of Animal Sciences, University of Florida, Gainesville, FL, USA; m Monitoring and Surveillance Unit, Department of Nutrition and Food Safety, WHO, Geneva, Switzerland; n School of Environmental Health Sciences, College of Health and Medical Sciences of Haramaya University, Dire Dawa, Ethiopia; o Department of Epidemiology and Health Economics, University Medical Center Utrecht, Utrecht University, Utrecht, Netherlands; p Centre for Complex System Studies, Utrecht University, Utrecht, Netherlands; q Interdisciplinary Center for the Mathematical Modeling of Infectious Disease Dynamics, University of Münster, Münster, Germany; r Delft Institute of Applied Mathematics, Faculty of Electrical Engineering, Mathematics and Computer Science, Delft University of Technology, Delft, Netherlands; s George Washington University, Milken Institute School of Public Health, Washington, DC, USA; t US Department of Agriculture, Economic Research Service, Washington, DC, USA; u National Institute for Public Health and the Environment, Center for Infectious Disease Control, Bilthoven, Netherlands; v Faculty of Veterinary Medicine, Utrecht University, Utrecht, Netherlands; w Department of Tropical Medicine, Faculty of Medicine, Khon Kaen University, Khon Kaen, Thailand; x National Institute of Health Sciences, Division of Food Safety Information, Kawasaki, Japan; y Reproductive, Maternal, Newborn and Child Health Research Unit, Research Institute of the Centre of Expertise and Biological Diagnostic of Cameroon, Yaounde, Cameroon; z Antimicrobial Research Unit, School of Health Sciences, College of Health Sciences, University of KwaZulu-Natal, Durban, South Africa; aa Infection and Global Health Division, School of Medicine, University of St Andrews, St Andrews, UK; ab Microbiology Laboratory, China National Center for Food Safety Risk Assessment, Beijing, China; ac Saudi Food and Drug Authority, Riyadh, Saudi Arabia; ad Research Center for Public Health and Nutrition, National Research and Innovation Agency, Soekarno Science and Technology Area, Bogor, Indonesia; ae Department of Molecular Biomedicine, CINVESTAV-IPN, Mexico City, Mexico; af Indian Council of Agricultural Research, Krishi Bhavan, New Delhi, India; ag Abu Dhabi Agriculture and Food Safety Authority, Abu Dhabi, United Arab Emirates; ah Department of Agricultural and Resource Economics, University of Maryland, College Park, MD, USA; ai Department of Food, Agricultural & Resource Economics, University of Guelph, Guelph, ON, Canada

## Abstract

**Background:**

Foodborne diseases are important causes of illness and death. The first estimates of their burden were published by WHO in 2015. We updated WHO estimates of the global, regional, subregional, and national foodborne disease burden caused by 42 infectious and chemical hazards in 2021, including time trends for 2000–21.

**Methods:**

We provide a high-level summary of foodborne disease burden, expressed as incidence, deaths, and disability-adjusted life-years (DALYs). Data for burden estimation were provided from a WHO-commissioned series of systematic reviews on the incidence, aetiology, sequelae, and case fatality or mortality of the hazards. Data were analysed using hierarchical meta-regression modelling with geographical clustering and a global linear time trend, disease-specific computational models, and uncertainty propagation through Monte Carlo simulations to calculate 95% uncertainty intervals. Attribution to foodborne transmission was principally based on a structured expert judgement process. Economic impact was measured as lost productivity.

**Findings:**

For 2021, foodborne transmission of the 42 hazards caused 866 million (95% uncertainty interval 680–1090) illnesses, 1·52 million (0·783–2·51) deaths, and 57·1 million (39·4–81·1) DALYs. Inorganic arsenic, lead, and non-typhoidal Salmonella enterica (diarrhoeal and invasive disease) resulted in the most DALYs. The greatest burden of foodborne disease was in the African and South-East Asia regions. The incidence in children younger than 5 years was 2·7 times higher than in people aged 5 years or older, resulting in 4·3 times the rate of DALYs. The total burden from all hazards decreased over time. In 2021, these 42 hazards resulted in productivity losses of US$310 billion in nominal terms, and US$647 billion after adjusting for purchasing power parity.

**Interpretation:**

Foodborne diseases causes a burden similar to that from tuberculosis, HIV and AIDS, or malaria. The high burden of both communicable and non-communicable foodborne diseases requires countries to prioritise developing strategies to improve the safety of the food supply.

**Funding:**

WHO.

## Introduction

Access to safe nutritious food is an important determinant of health. Unsafe food causes a substantial global population health burden, as well as being a barrier to achieving the UN 2030 Sustainable Development Goals.^1^ Global-level and national-level data on foodborne disease burden over time are needed to improve and monitor the effectiveness of food safety systems, prevent foodborne disease, and improve public health.^2^ Economic estimates are needed to justify and evaluate investments in food safety.^3^

Foodborne disease is principally caused by eating food contaminated with biological or chemical hazards. WHO and the Foodborne Disease Burden Epidemiology Reference Group (FERG, 2007–15) reported that one in ten people globally had foodborne disease each year (reference year 2010), comprising 600 million cases of illness and 420 000 deaths, leading to a disease burden of 33 million disability-adjusted life-years (DALYs).^4^ Although acknowledged as an underestimate, the public health impact of foodborne diseases was in the same order as other major global public health issues, such as malaria, HIV, and tuberculosis. The World Bank subsequently converted these 2010 estimates into economic terms, highlighting that unsafe food costs national governments in low-income and middle-income countries at least US$110 billion in lost productivity and medical expenses.^3^ Additional burden estimates, for dietary exposure to four metals, were published subsequently, adding an estimated 1 million illnesses, 56 000 deaths, and 9 million DALYs.^5^

**Research in context**

**Evidence before this study**

Access to safe, nutritious food is an important determinant of health. Unsafe food causes foodborne illness through contamination with biological or chemical hazards. Although individual countries have estimated the national burden of foodborne disease to support prevention and intervention strategies, the first estimates of the global burden of foodborne disease were reported by WHO in 2015. These estimates covered 31 hazards and demonstrated that globally one in ten people had foodborne disease each year, resulting in up to 600 million illnesses, 420 000 deaths, and 33 million healthy life years lost in 2010. The burden was highest in Africa and South-East Asia and lowest in high-income countries. That study identified gaps in evidence and methodological limitations, and needed updating due to advances in data and methodology.

**Added value of this study**

This study synthesises the new 2026 edition of the WHO estimates of the global burden of foodborne diseases due to 42 enteric (diarrhoeal and invasive non-diarrhoeal), parasitic, and chemical hazards, providing subregional patterns, comparative ranking across all hazards, and global estimates of lost productivity. For 2021, foodborne transmission of the 42 hazards caused 866 million illnesses, 1·52 million deaths, and 57·1 million disability-adjusted life-years. This study shows, for the first time, the considerable burden from non-communicable diseases, in particular cardiovascular diseases, cancer, and intellectual disability resulting from dietary exposure to metals, particularly inorganic arsenic and lead. Despite a global decline in overall burden from all hazards for the period 2000–21, major inequalities in burden across regions persist. To address the economic burden from foodborne disease, which is important to policy makers, this report includes an estimate of lost productivity expressed in monetary terms, derived from the estimates of disability-adjusted life-years. In 2021, these 42 hazards resulted in productivity losses of US$310 billion in nominal terms and US$647 billion after adjusting for purchasing power parity.

**Implications of all the available evidence**

Foodborne disease remains an important public health problem globally, causing significant economic losses. The burden is similar to other important diseases, such as tuberculosis, HIV, and malaria. Despite the estimation that foodborne exposure to inorganic arsenic and lead are the leading hazards in terms of burden, it is important to manage the burden from microbial hazards through well understood interventions such as pasteurisation of milk and interruption of parasitic lifecycles. Providing foodborne disease estimates at global, regional, and national levels can inform countries’ priorities and help them to tailor actions and allocate resources to improve food safety and monitor the impact of interventions.

In 2020, the Seventy-third World Health Assembly adopted a resolution “Strengthening efforts on food safety” (WHA73.5).^6^ This resolution mandated WHO to regularly monitor the global burden of foodborne and zoonotic diseases at national, regional, and global levels. It also specifically requested WHO update burden estimates by 2025. In addition, in 2022 WHO member states committed to the WHO global strategy for food safety for 2022–30.^1^

We report aggregated updated WHO estimates for the burden of foodborne disease from 42 hazards, alongside subregional estimates of incidence, mortality, and burden, and show the global productivity impact of foodborne diseases. This study also provides trends of the total burden of foodborne diseases for the period 2000–21 from all the hazards. National estimates were made available to feedback from WHO member states through official country consultation. These aggregated results are collated from results for individual hazard groups, and associated methodologies reported in accompanying papers.^7^, ^8^, ^9^, ^10^, ^11^, ^12^

## Methods

### Study overview

In 2021, the WHO Department of Nutrition and Food Safety reconvened the FERG to advise and support WHO's efforts to update estimates of the burden of foodborne diseases. This technical advisory group appointed for 2021–25 comprised 26 independent experts with specialised knowledge in various domains, including foodborne diseases, epidemiology, data science, biostatistics, microbiology, parasitology, chemical and microbial risk assessment, disease burden estimation, and health economics. Subgroups (task forces) of FERG were established to address hazard types (chemical, enteric, and parasitic; appendix p 3), source attribution, and computation methodology. This technical leadership was complemented by a large network of about 200 scientists worldwide who participated in systematic reviews, technical discussions, and structured expert judgment. These scientists are acknowledged in the associated papers^7^, ^8^, ^9^, ^10^, ^11^, ^12^ (two of which are preprints^10^, ^12^).

The methodology and results are described in full detail in separate papers.^7^, ^8^, ^9^, ^10^, ^11^, ^12^ Here, we provide a high-level summary of methodology and results (appendix p 5). As with the previous estimates, we chose a hazard-based and incidence-based approach, with the burden expressed as incidence, mortality, and DALYs because the rationale for this approach remains valid.^13^ In the incidence-based approach, all health outcomes, including those in future years, are assigned to the initial event (eg, exposure to a certain hazard). This approach therefore reflects the future burden of disease resulting from current events.

The 2010 estimates included 31 hazards. All of these were retained, apart from three bacterial toxins (*Clostridium perfringens*, *Bacillus cereus*, and *Staphylococcus aureus*) that had minimal burden and were previously estimated for high-income countries only.^14^

The 2010 estimates were acknowledged as limited by including only a subset of the potential foodborne hazards.^4^ In an effort to provide a more complete estimation of the foodborne disease burden, each task force reviewed these and potential additional hazards according to agreed criteria. Hazard selection processes are detailed in separate papers describing the burden from diarrhoeal, invasive (non-diarrhoeal) enteric, parasitic, and chemical hazards.^7^, ^8^, ^9^, ^11^ Important hazard additions were *Trypanosoma cruzi* (causing Chagas disease), *Cyclospora cayetanensis*, enteroaggregative *Escherichia coli*, rotavirus, aflatoxin M1, and four metals (inorganic arsenic, methylmercury, lead, and cadmium). Health states resulting from exposure to the hazards were also reviewed. The chosen 42 hazards and 47 health states are shown in the appendix (p 3).

For regional analysis and to support imputations for countries with missing data, the six WHO regions were subdivided into 17 subregional clusters (appendix p 4) based on World Bank income classifications.^12^ In accordance with resolution WHA78.25 (2025), Indonesia was reassigned to the WHO Western Pacific region as of May 27, 2025. In this instance, data analysis had already been completed with data pertaining to Indonesia included in the South-East Asia regional aggregates. WHO regional comparisons must be considered with caution.

Reporting guidelines for global health (GATHER) and burden of disease (STROBOD) studies were followed for individual hazard group estimates and for this high-level summary.^7^, ^8^, ^9^, ^11^, ^15^, ^16^

### Burden estimation

To provide data for burden estimation, WHO commissioned systematic reviews through an open call for applications and selection process. These reviews principally collated data on the incidence of the health states according to hazard-specific outcome trees, as well as reviewing evidence for linkages between some hazards and health outcomes. PROSPERO protocols for the reviews are referenced in the accompanying papers.^7^, ^8^, ^9^, ^11^

The following two approaches, which are described in greater detail elsewhere, were used to estimate burden for the 14 diarrhoeal hazards.^8^ In all subregional cluster A countries (ie, region of the Americas A, Eastern Mediterranean region A, European region A, and Western Pacific region A), national data on population-level incidence or mortality from diarrhoeal diseases were obtained from an updated scoping review of studies, with additional data identified during WHO's country consultation process. For all other countries, the incidence of disease caused by the 14 diarrhoeal hazards was estimated by modelling proportions of the diarrhoeal disease incidence estimates from the Global Burden of Disease Study (GBD) 2021,^17^ and diarrhoeal mortality estimates from the WHO's global health estimates, for the years 2000 to 2021.^8^, ^18^, ^19^ For seven invasive (non-diarrhoeal) enteric hazards and one foodborne intoxication, estimates were derived from national or regional estimates of incidence obtained through systematic reviews, and from data from the Institute of Health Metrics and Evaluation (IHME).^7^

For invasive (non-diarrhoeal) parasitic hazards, incidence data from systematic reviews provided information on 11 of the 12 parasitic diseases under consideration. For Chagas disease, for which no systematic review was commissioned, relevant data were provided by the IHME.^11^

For chemical hazards, burden estimates were developed using categorical or foodborne attribution and risk assessment approaches.^9^ Population attributable fractions were estimated for aflatoxins and inorganic arsenic to estimate the contribution from dietary exposure to the diseases under consideration. Incidence of diseases induced by cadmium, dioxin, and methylmercury were estimated by risk assessment approaches combining dose–response relationship with exposure data. Systematic reviews provided available data on the incidence and mortality for konzo and peanut allergy. Estimates of the global burden of foodborne disease caused by lead exposure were based on IHME's estimates of global burden of cardiovascular diseases and intellectual disability due to lead (2000–21).^20^

### Attribution of incidence to foodborne transmission

We applied the same methodology as for the previous estimates for source attribution using structured expert judgment at the subregional level, guided by the FERG task forces.^10^, ^21^ For some hazards, approaches based on empirical data were considered to be more appropriate. About 150 experts provided more than 1400 tailored hazard and subregion sets of assessments with uncertainty for major pathways and for source attribution for specific food groups. Individual uncertainty distributions were mathematically aggregated using a validated approach, and samples and estimates were obtained from the resulting distributions. Details of the process and results are reported elsewhere.^10^

### Economics

The direct economic impacts of illness include medical expenses, the value of lost time (lost productivity), and the value people place on deaths. Here, economic impact was measured as lost productivity (gross national income per capita times DALYs). This is a conservative measure of economic impact and is the same as used to value the 2010 burden estimates.^3^ Estimates are provided by region and by age (all ages, age <5 years, and age ≥5 years).

### Data analysis

Data cleaning is described in the appendix (p 13). We used a standardised data analysis pipeline to address data gaps and estimate cases, deaths, and DALYs by country and year. Briefly, we used a Bayesian hierarchical meta-regression model with geographical clustering and a global time trend to smooth and impute country-specific and year-specific input data. Uncertainty in input parameters was propagated for estimates of illnesses, deaths, and DALYs through 500 Monte Carlo simulations following disease-specific computational disease models defined by incidence and probability parameters. Demographic data were sourced from the UN World Population Prospects 2024 Revision.^22^ To impute incidence values for countries with missing data, a hierarchical approach used data from the same subregion, broader region, and global year-specific estimates according to availability. 95% uncertainty intervals (UIs) were defined as the 2·5th and 97·5th percentiles of the joint uncertainty distribution of the estimated outcomes.

Details on all analytical steps, including mathematical formulae, data cleaning and pre-processing, and specific models used, including their selection and evaluation, are given elsewhere.^12^

Calculations were performed at the national level and aggregated for subregional, regional, and global totals. In line with current global burden of disease assessments, no age weighting or time discounting was applied. Most of the disability weights were taken from GBD sources.^23^ Analyses were performed in R (version 4.5.0 and higher).

### Role of the funding source

WHO funded systematic reviews and the structured expert judgment study that provided data for this analysis, expert consultation (University of Florida, Gainesville, FL, USA), and Sciensano (CdB and LV), which did the computations for this work. The economic estimates were funded by the scientists involved (SHo, SJ, and SHe). WHO managed the overall project and the country consultation process that identified additional data, although each task force determined whether data met inclusion criteria before incorporating them in the analyses. WHO cleared the paper in terms of methods and approach. WHO staff (YM, CR, EB, RK, and LI) participated in drafting the manuscript and the decision to submit it for publication.

## Results

For this paper, we present results for 2021 at the global, regional, subregional, and national levels. Detailed national-level estimates are available in WHO's Global Health Observatory,^24^ with time trends for all hazards available via WHO's interactive dashboard.^25^

In 2021, 2·56 billion illnesses were estimated to have been caused by the 42 hazards in this study. Of these, 866 million (95% UI 680–1090) were attributed to foodborne transmission. Hazards causing diarrhoea caused approximately 666 million (483–884) of these foodborne illnesses, while invasive enteric and parasitic disease hazards caused approximately 194 million (145–238) illnesses.^7^, ^8^, ^11^ Chemical foodborne hazards were estimated to have caused 6·26 million (3·36–10·3) illnesses.^9^ Overall, the 42 foodborne hazards caused an estimated 1·52 million (0·783–2·51) deaths in 2021. The total burden of foodborne disease in 2021 was estimated at 57·1 million (39·4–81·1) DALYs, from 8·36 million (6·18–11·4) years lived with disability (YLDs), and 48·8 million (31·8–71·8) years of life lost (YLLs; table 1).

**Table 2:** Mean rates of foodborne disability-adjusted life-years per 100 000 population by WHO subregion, 2021

			**AFR AB**	**AFR C**	**AFR D**	**AMR A**	**AMR B**	**AMR C**	**SEAR B**	**SEAR CD**	**EUR A**	**EUR B**	**EUR C**	**EMR A**	**EMR BC**	**EMR D**	**WPR A**	**WPR B**	**WPR C**
Total			617·3 (421·2 to 932·6)	1261·3 (875·2 to 1786·0)	1150·1 (849·7 to 1535·7)	195·2 (106·5 to 378·1)	390·0 (266·1 to 637·7)	638·4 (415·1 to 1069·3)	750·3 (401·4 to 1323·7)	1264·0 (655·1 to 2256·2)	155·2 (85·4 to 309·9)	288·6 (117·4 to 624·1)	356·2 (170·9 to 758·6)	109·8 (63·6 to 226·7)	585·4 (397·9 to 917·1)	704·1 (427·4 to 1168·2)	179·0 (110·2 to 283·4)	478·7 (190·0 to 1091·0)	811·2 (392·8 to 1587·1)
Diarrhoeal disease hazards			361·9 (226·5 to 590·2)	1048·9 (690·2 to 1575·9)	843·8 (579·9 to 1209·1)	24·5 (11·1 to 68·5)	38·7 (15·4 to 111·1)	104·1 (55·6 to 192·3)	96·2 (55·8 to 153·6)	206·0 (119·6 to 319·0)	27·6 (14·1 to 51·5)	19·4 (6·88 to 51·1)	30·0 (14·7 to 64·5)	26·7 (10·8 to 85·4)	240·4 (147·8 to 372·3)	421·1 (215·6 to 682·5)	12·0 (5·88 to 30·5)	9·08 (3·77 to 28·5)	84·7 (44·3 to 138·7)
	Viruses		54·1 (8·93 to 114·2)	190·4 (29·9 to 428·1)	125·0 (18·8 to 275·9)	3·91 (0·780 to 10·4)	2·26 (0·503 to 4·85)	13·5 (2·96 to 30·0)	21·0 (4·16 to 52·8)	28·4 (3·71 to 72·6)	5·94 (0·912 to 14·8)	1·13 (0·306 to 3·64)	3·36 (0·799 to 9·46)	3·41 (0·464 to 9·13)	12·0 (2·62 to 37·9)	27·5 (5·25 to 91·6)	2·70 (0·539 to 5·71)	2·20 (0·568 to 4·54)	29·3 (5·92 to 67·9)
		Norovirus	13·4 (1·94 to 38·8)	34·5 (5·93 to 87·6)	30·3 (5·09 to 78·3)	3·60 (0·491 to 10·2)	1·30 (0·158 to 3·41)	7·57 (1·03 to 20·9)	9·03 (1·19 to 23·3)	12·5 (0·348 to 35·4)	5·70 (0·589 to 14·7)	0·574 (0·096 to 1·52)	1·50 (0·170 to 3·78)	3·34 (0·457 to 9·11)	9·51 (2·31 to 22·9)	20·5 (3·40 to 51·8)	2·25 (0·301 to 5·29)	1·05 (0·210 to 1·97)	12·0 (1·55 to 28·8)
		Rotavirus	40·7 (0·827 to 99·8)	155·9 (3·08 to 391·7)	94·7 (1·56 to 238·0)	0·31 (0·011 to 0·966)	0·966 (0·031 to 2·85)	5·91 (0·199 to 17·2)	11·9 (0·075 to 40·8)	15·9 (0·051 to 52·6)	0·240 (0·018 to 1·14)	0·559 (0·026 to 2·97)	1·86 (0·154 to 7·90)	0·067 (<0·001 to 0·683)	2·45 (<0·001 to 26·8)	7·07 (<0·001 to 72·0)	0·449 (0·013 to 1·69)	1·15 (0·047 to 3·26)	17·4 (0·766 to 52·6)
	Bacteria		275·4 (153·4 to 489·9)	701·7 (454·8 to 1082·8)	639·8 (414·5 to 967·1)	20·3 (8·14 to 64·0)	34·8 (12·2 to 107·0)	83·2 (39·4 to 166·9)	65·2 (32·9 to 113·6)	161·5 (91·9 to 269·9)	21·5 (10·8 to 45·7)	17·6 (5·37 to 49·2)	25·1 (10·9 to 59·8)	23·1 (7·98 to 80·3)	213·3 (124·0 to 341·5)	362·3 (176·9 to 603·6)	9·08 (3·85 to 28·1)	6·56 (2·16 to 26·2)	50·4 (26·7 to 86·1)
		*Campylobacter jejuni* or Campylobacter coli*	45·3 (14·4 to 91·1)	93·8 (27·7 to 188·0)	85·8 (26·2 to 161·6)	2·89 (1·33 to 5·53)	4·07 (1·21 to 7·85)	19·8 (6·93 to 40·8)	11·8 (4·19 to 25·8)	31·0 (12·1 to 63·1)	5·29 (2·08 to 9·57)	1·28 (0·580 to 2·54)	3·60 (1·31 to 7·20)	3·66 (0·983 to 11·9)	54·0 (18·6 to 113·9)	72·7 (25·7 to 145·9)	1·84 (0·891 to 3·46)	1·06 (0·453 to 2·05)	15·0 (5·92 to 29·1)
		Enteroaggregative *Escherichia coli*†	11·7 (−21·5 to 59·4)	28·3 (−52·0 to 148·1)	30·0 (−53·7 to 135·7)	0 (0 to 0)	1·30 (−2·33 to 5·64)	5·86 (−9·45 to 29·5)	6·06 (−9·90 to 28·8)	10·7 (−16·3 to 44·6)	0 (0 to 0)	0·269 (−0·440 to 1·29)	1·06 (−1·82 to 5·26)	0 (0 to 0)	17·4 (−31·6 to 76·8)	31·8 (−52·4 to 136·7)	0 (0 to 0)	0·089 (−0·143 to 0·485)	4·12 (−6·14 to 21·2)
		Enteropathogenic *Escherichia coli*†	16·6 (−1·46 to 48·8)	20·2 (−2·44 to 58·5)	45·6 (−6·86 to 121·2)	0 (0 to 0)	0·490 (−0·016 to 1·54)	2·65 (−0·217 to 8·41)	2·50 (−0·137 to 8·15)	3·07 (−0·187 to 9·52)	0 (0 to 0)	0·292 (0·005 to 0·792)	0·857 (−0·091 to 2·77)	0 (0 to 0)	12·8 (−1·69 to 38·8)	22·5 (−3·00 to 69·2)	0 (0 to 0)	0·069 (−0·001 to 0·222)	1·49 (−0·143 to 5·14)
		Enterotoxigenic *Escherichia coli*	41·4 (7·74 to 92·5)	112·6 (46·7 to 216·6)	105·1 (41·7 to 196·1)	0·018 (0·003 to 0·060)	1·65 (0·356 to 3·90)	6·21 (0·568 to 17·5)	12·9 (3·63 to 31·2)	11·8 (3·52 to 30·2)	0·018 (0·002 to 0·060)	0·885 (0·296 to 1·93)	1·98 (0·522 to 4·76)	0·024 (0·004 to 0·078)	34·0 (11·6 to 75·2)	67·2 (22·1 to 148·3)	0·009 (<0·001 to 0·032)	0·154 (0·018 to 0·393)	3·11 (0·367 to 8·80)
		Shiga toxin-producing *Escherichia coli*	26·3 (6·23 to 74·3)	73·0 (21·5 to 195·9)	46·5 (12·5 to 113·3)	1·79 (0·784 to 3·38)	3·30 (0·706 to 8·48)	8·44 (1·83 to 24·1)	7·35 (0·886 to 25·0)	18·1 (4·67 to 46·4)	1·08 (0·137 to 3·04)	2·25 (0·114 to 8·67)	2·23 (0·153 to 9·01)	1·58 (0·581 to 3·39)	19·7 (6·24 to 53·3)	37·6 (11·2 to 101·2)	0·490 (0·111 to 1·26)	0·130 (0·027 to 0·448)	2·84 (0·310 to 9·30)
		Non-typhoidal *Salmonella enterica*‡	77·2 (16·2 to 269·9)	243·9 (98·9 to 534·7)	183·1 (75·0 to 439·4)	15·4 (4·02 to 59·4)	21·8 (2·63 to 94·3)	29·0 (6·16 to 101·6)	14·8 (4·88 to 40·8)	23·2 (8·55 to 55·3)	15·1 (5·76 to 38·7)	12·3 (1·58 to 43·6)	14·1 (2·85 to 46·1)	17·8 (3·84 to 75·6)	26·1 (5·87 to 105·7)	67·7 (17·6 to 204·4)	6·69 (1·86 to 25·2)	4·73 (0·737 to 24·2)	15·7 (5·42 to 44·5)
		*Shigella* spp	55·6 (21·9 to 103·4)	112·8 (47·8 to 207·0)	117·8 (49·9 to 222·1)	0·128 (0·015 to 0·385)	2·22 (0·387 to 6·51)	10·0 (0·978 to 28·1)	9·85 (0·546 to 33·5)	36·3 (4·56 to 89·2)	0·048 (0·008 to 0·145)	0·394 (0·091 to 0·946)	1·29 (0·242 to 3·50)	0·072 (0·004 to 0·271)	33·1 (0·874 to 77·9)	44·9 (1·95 to 169·7)	0·055 (0·004 to 0·182)	0·331 (0·045 to 0·864)	7·32 (0·337 to 20·8)
		*Vibrio cholerae*	1·27 (0·145 to 3·61)	17·0 (1·85 to 42·9)	25·9 (3·14 to 72·8)	0 (0 to 0)	0 (0 to 0)	1·31 (0·155 to 4·18)	0 (0 to 0)	27·4 (3·12 to 81·6)	0 (0 to 0)	0 (0 to 0)	0 (0 to 0)	0 (0 to 0)	16·1 (2·77 to 49·3)	17·9 (3·44 to 59·5)	0 (0 to 0)	0 (0 to 0)	0·768 (0·050 to 2·39)
	Protozoa		32·4 (13·2 to 65·4)	156·8 (55·3 to 360·7)	79·1 (37·2 to 147·8)	0·353 (0·050 to 0·812)	1·67 (0·566 to 3·59)	7·36 (2·40 to 20·3)	9·98 (2·62 to 29·6)	16·1 (5·09 to 36·5)	0·165 (0·038 to 0·429)	0·648 (0·192 to 1·45)	1·59 (0·434 to 4·16)	0·216 (0·041 to 0·523)	15·2 (4·35 to 34·8)	31·3 (9·11 to 77·3)	0·200 (0·047 to 0·444)	0·321 (0·054 to 1·02)	5·03 (0·974 to 14·3)
		*Cryptosporidium* spp	16·2 (3·78 to 38·0)	62·7 (15·1 to 158·2)	46·1 (14·2 to 90·1)	0·296 (0·014 to 0·734)	0·483 (0·032 to 1·46)	1·31 (0·084 to 4·79)	1·53 (0·150 to 4·99)	4·99 (0·616 to 13·6)	0·125 (0·017 to 0·366)	0·155 (0·012 to 0·577)	0·574 (0·034 to 2·58)	0·161 (0·022 to 0·413)	6·71 (0·757 to 20·7)	9·73 (0·846 to 33·7)	0·162 (0·021 to 0·409)	0·037 (0·004 to 0·113)	1·90 (0·187 to 6·24)
		*Cyclospora cayetanensis*†	12·3 (1·93 to 39·4)	89·1 (14·4 to 293·0)	26·9 (5·05 to 89·1)	0·006 (0·001 to 0·018)	0·822 (0·136 to 2·85)	4·40 (0·617 to 16·0)	6·43 (0·801 to 23·7)	4·78 (0·788 to 19·0)	0·007 (0·001 to 0·021)	0·360 (0·042 to 1·18)	0·729 (0·085 to 2·53)	0·006 (<0·001 to 0·017)	6·16 (1·00 to 18·1)	18·4 (3·50 to 58·1)	0·006 (<0·001 to 0·019)	0·235 (0·009 to 0·901)	2·46 (0·087 to 10·5)
		*Entamoeba histolytica*	3·45 (0·080 to 18·1)	4·60 (0·129 to 18·3)	5·32 (0·214 to 20·6)	0·006 (<0·001 to 0·035)	0·291 (0·010 to 1·09)	1·55 (0·115 to 5·37)	1·90 (0·044 to 6·97)	5·85 (0·179 to 20·2)	0·005 (<0·001 to 0·024)	0·078 (0·002 to 0·280)	0·240 (0·008 to 1·08)	0·004 (<0·001 to 0·023)	1·80 (0·066 to 8·97)	2·89 (0·041 to 12·0)	0·004 (<0·001 to 0·025)	0·044 (0·001 to 0·184)	0·554 (0·014 to 2·46)
		*Giardia duodenalis*	0·470 (0·022 to 1·78)	0·486 (0·029 to 1·65)	0·750 (0·026 to 2·86)	0·045 (0·002 to 0·142)	0·072 (0·003 to 0·337)	0·090 (0·002 to 0·414)	0·124 (0·005 to 0·532)	0·469 (0·018 to 1·57)	0·027 (0·001 to 0·093)	0·055 (0·002 to 0·239)	0·044 (0·001 to 0·196)	0·045 (0·001 to 0·190)	0·531 (0·020 to 2·23)	0·282 (0·011 to 1·24)	0·027 (0·002 to 0·096)	0·005 (<0·001 to 0·018)	0·120 (0·005 to 0·411)
Invasive disease hazards			129·0 (67·3 to 249·9)	100·0 (54·6 to 194·8)	186·7 (118·3 to 298·5)	26·2 (12·5 to 44·6)	155·6 (109·0 to 224·9)	270·0 (184·5 to 380·0)	136·5 (58·8 to 283·6)	213·9 (113·0 to 364·0)	15·2 (10·3 to 21·9)	17·6 (11·6 to 27·3)	19·8 (11·1 to 39·5)	21·4 (12·6 to 34·1)	164·5 (84·6 to 292·4)	87·6 (48·1 to 153·6)	16·9 (8·95 to 31·3)	60·7 (30·1 to 121·5)	321·3 (87·4 to 937·8)
	Viruses		1·26 (0·400 to 2·28)	4·16 (1·17 to 8·39)	7·01 (1·68 to 15·0)	0·338 (0·101 to 0·603)	1·06 (0·247 to 2·06)	2·19 (0·524 to 4·17)	8·16 (2·34 to 17·2)	35·0 (7·72 to 73·1)	0·071 (0·002 to 0·141)	0·812 (0·047 to 1·78)	1·49 (0·255 to 3·08)	1·33 (0·567 to 2·41)	12·2 (2·15 to 31·4)	19·0 (3·41 to 43·9)	0·042 (0·008 to 0·074)	0·956 (0·180 to 1·82)	2·63 (0·572 to 5·17)
		Hepatitis A virus	1·26 (0·400 to 2·28)	4·16 (1·17 to 8·39)	7·01 (1·68 to 15·0)	0·338 (0·101 to 0·603)	1·06 (0·247 to 2·06)	2·19 (0·524 to 4·17)	8·16 (2·34 to 17·2)	35·0 (7·72 to 73·1)	0·071 (0·002 to 0·141)	0·812 (0·047 to 1·78)	1·49 (0·255 to 3·08)	1·33 (0·567 to 2·41)	12·2 (2·15 to 31·4)	19·0 (3·41 to 43·9)	0·042 (0·008 to 0·074)	0·956 (0·180 to 1·82)	2·63 (0·572 to 5·17)
	Bacteria		33·2 (10·6 to 69·3)	54·5 (17·4 to 147·0)	94·2 (42·4 to 193·1)	3·13 (1·58 to 5·62)	7·79 (3·03 to 22·9)	11·5 (4·81 to 28·6)	71·7 (18·3 to 153·4)	118·4 (31·6 to 250·2)	5·35 (3·77 to 7·47)	2·61 (0·934 to 6·25)	3·47 (0·770 to 10·2)	7·56 (2·75 to 16·9)	123·8 (47·5 to 247·9)	49·1 (18·6 to 106·2)	3·12 (1·22 to 7·18)	2·88 (0·952 to 6·13)	46·6 (17·9 to 100·0)
		*Brucella* spp	0·002 (<0·001 to 0·009)	0·043 (0·005 to 0·161)	0·006 (<0·001 to 0·036)	0·002 (<0·001 to 0·004)	0·029 (0·005 to 0·102)	0·004 (<0·001 to 0·015)	0·008 (<0·001 to 0·054)	0·002 (<0·001 to 0·011)	0·007 (<0·001 to 0·020	0·187 (0·019 to 0·917)	0·124 (0·004 to 0·609)	0·283 (0·037 to 1·03)	0·288 (0·063 to 0·836)	0·787 (0·028 to 4·88)	0·002 (<0·001 to 0·010)	0·097 (0·024 to 0·241)	0·015 (<0·001 to 0·107)
		*Listeria monocytogenes*	6·50 (0·698 to 28·5)	3·72 (0·305 to 18·8)	3·72 (0·305 to 18·8)	2·89 (1·38 to 5·33)	3·36 (0·353 to 16·7)	3·37 (0·354 to 16·8)	2·42 (0·372 to 9·24)	2·42 (0·372 to 9·25)	5·28 (3·72 to 7·41)	1·48 (0·301 to 4·72)	2·61 (0·357 to 9·24)	2·42 (0·371 to 9·23)	2·42 (0·373 to 9·26)	2·43 (0·373 to 9·28)	2·79 (0·981 to 6·70)	0·036 (0·011 to 0·091)	1·65 (0·180 to 6·56)
		*Mycobacterium bovis, Mycobacterium caprae,* or *Mycobacterium orygis*	26·4 (6·24 to 55·4)	17·5 (4·49 to 33·5)	22·7 (10·3 to 41·5)	0·228 (0·061 to 0·565)	4·01 (1·56 to 8·42)	5·92 (1·79 to 14·2)	14·4 (2·17 to 36·2)	10·6 (0·938 to 34·0)	0·056 (0·007 to 0·185)	0·642 (0·071 to 2·31)	0·729 (0·064 to 2·81)	0·449 (0·031 to 1·66)	19·6 (2·30 to 56·3)	23·4 (5·45 to 70·9)	0·325 (0·028 to 1·09)	1·20 (0·146 to 3·66)	11·1 (1·85 to 28·0)
		*Salmonella enterica* serotype Paratyphi A, B, and C	<0·001 (<0·001 to 0·008)	0·051 (<0·001 to 0·475)	0·009 (<0·001 to 0·079)	0·005 (0·001 to 0·011)	0·006 (0·001 to 0·012)	0·283 (0·033 to 0·760)	0·332 (0·019 to 1·00)	13·2 (1·40 to 34·1)	0·001 (<0·001 to 0·004)	0·004 (<0·001 to 0·011)	<0·001 (<0·001 to 0·001)	0·059 (0·016 to 0·156)	29·9 (11·7 to 63·4)	0·178 (0·054 to 0·400)	0·001 (<0·001 to 0·003)	0·127 (0·008 to 0·386)	2·01 (0·565 to 5·10)
		*Salmonella enterica* serotype Typhi	0·311 (0·031 to 1·50)	33·2 (3·05 to 120·2)	67·8 (21·6 to 158·6)	0·002 (<0·001 to 0·004)	0·395 (0·113 to 0·637)	1·91 (0·506 to 4·18)	54·5 (4·61 to 135·2)	92·2 (8·51 to 216·0)	0·005 (<0·001 to 0·010)	0·300 (0·049 to 0·882)	0·005 (<0·001 to 0·015)	4·35 (0·178 to 11·6)	71·6 (13·1 to 167·4)	22·4 (4·24 to 53·1)	0·007 (<0·001 to 0·016)	1·42 (0·222 to 3·57)	31·8 (7·48 to 81·2)
	Parasites		94·6 (39·5 to 203·0)	41·3 (24·6 to 68·6)	85·5 (51·1 to 137·7)	22·8 (9·30 to 41·5)	146·8 (101·1 to 213·8)	256·4 (171·7 to 365·5)	56·6 (23·1 to 187·4)	60·4 (41·9 to 98·9)	9·75 (5·26 to 15·8)	14·2 (8·31 to 23·7)	14·8 (7·58 to 29·7)	12·5 (6·24 to 21·8)	28·5 (16·4 to 47·0)	19·5 (10·5 to 30·7)	13·7 (6·46 to 27·3)	56·9 (27·2 to 117·2)	272·1 (45·4 to 892·9)
		*Toxoplasma gondii*§	7·46 (3·22 to 13·7)	11·4 (5·69 to 19·5)	11·3 (5·68 to 19·8)	21·3 (7·93 to 39·7)	40·2 (19·1 to 70·6)	47·4 (22·0 to 91·8)	10·5 (4·53 to 18·9)	8·71 (1·75 to 18·4)	9·08 (4·67 to 15·2)	9·46 (5·32 to 15·5)	6·75 (3·69 to 11·3)	11·0 (5·09 to 19·9)	13·1 (6·75 to 20·7)	14·5 (7·29 to 24·4)	8·08 (3·37 to 17·8)	1·27 (0·572 to 2·44)	4·30 (1·83 to 8·41)
		*Trypanosoma cruzi*	0 (0 to 0)	0 (0 to 0)	0 (0 to 0)	0·909 (0·558 to 1·38)	77·5 (47·3 to 117·2)	140·6 (81·9 to 211·6)	0 (0 to 0)	0 (0 to 0)	0 (0 to 0)	0 (0 to 0)	0 (0 to 0)	0 (0 to 0)	0 (0 to 0)	0 (0 to 0)	0 (0 to 0)	0 (0 to 0)	0 (0 to 0)
		*Echinococcus granulosus*	1·22 (0·077 to 4·48)	1·73 (0·126 to 5·69)	2·27 (0·072 to 7·82)	0·209 (0·031 to 0·602)	0·842 (0·100 to 2·45)	0·136 (0·007 to 0·544)	0 (0 to 0)	0·406 (0·070 to 1·21)	0·288 (0·042 to 0·842)	2·12 (0·436 to 6·17)	4·29 (0·634 to 12·2)	0·309 (0·035 to 1·07)	2·19 (0·340 to 5·57)	0·911 (0·071 to 2·58)	0·100 (0·010 to 0·377)	4·52 (0·574 to 13·1)	0·255 (0·027 to 0·830)
		*Echinococcus multilocularis*	0 (0 to 0)	0 (0 to 0)	0 (0 to 0)	<0·001 (<0·001 to 0·002)	0 (0 to 0)	0 (0 to 0)	0 (0 to 0)	0·001 (<0·001 to 0·003)	0·041 (0·014 to 0·086)	0·249 (0·015 to 0·784)	0·120 (0·034 to 0·252)	0 (0 to 0)	<0·001 (<0·001 to 0·001)	<0·001 (<0·001 to <0·001)	0·008 (0·002 to 0·018)	0·557 (0 to 2·96)	<0·001 (0 to 0·005)
		*Taenia solium*	63·1 (24·5 to 114·8)	17·1 (6·55 to 35·6)	60·2 (27·8 to 103·6)	0·031 (0·005 to 0·109)	9·17 (5·31 to 14·7)	37·6 (13·4 to 74·1)	4·19 (1·18 to 10·7)	34·8 (24·6 to 47·3)	0·016 (0·005 to 0·037)	0·055 (0·016 to 0·130)	0·036 (0·010 to 0·097)	0 (0 to 0)	0 (0 to 0)	0 (0 to 0)	0 (0 to 0)	8·93 (2·61 to 21·2)	13·6 (3·88 to 31·2)
		*Ascaris* spp	4·76 (0·004 to 11·4)	4·93 (0·004 to 9·19)	4·74 (0·004 to 9·59)	0·075 (<0·001 to 0·220)	2·68 (0·013 to 5·69)	4·39 (0·045 to 9·52)	8·43 (3·55 to 14·4)	6·75 (3·02 to 9·75)	0 (0 to 0)	0·085 (<0·001 to 0·211)	0·929 (0·004 to 3·42)	0·120 (<0·001 to 0·381)	4·59 (0·020 to 10·6)	2·97 (0·012 to 8·78)	0·005 (<0·001 to 0·019)	0·886 (0·004 to 1·85)	6·46 (0·004 to 20·2)
		*Trichinella* spp	0·002 (<0·001 to 0·007)	0·002 (<0·001 to 0·006)	0·001 (<0·001 to 0·004)	0·001 (<0·001 to 0·003)	0·007 (0·002 to 0·024)	0·002 (<0·001 to 0·008)	0·003 (<0·001 to 0·012)	0·011 (<0·001 to 0·080)	0·002 (0·001 to 0·004)	0·008 (0·004 to 0·020)	0·002 (<0·001 to 0·006)	<0·001 (<0·001 to 0·002)	<0·001 (<0·001 to 0·001)	<0·001 (<0·001 to 0·002)	0·002 (<0·001 to 0·008)	<0·001 (<0·001 to 0·002)	0·004 (<0·001 to 0·015)
		*Clonorchis sinensis*	0 (0 to 0)	0 (0 to 0)	0 (0 to 0)	0 (0 to 0)	0 (0 to 0)	0 (0 to 0)	0 (0 to 0)	0 (0 to 0)	0 (0 to 0)	<0·001 (<0·001 to 0·004)	0 (0 to 0)	0 (0 to 0)	0 (0 to 0)	0 (0 to 0)	4·67 (0·770 to 14·7)	32·7 (8·15 to 91·1)	173·1 (2·79 to 738·1)
		*Fasciola* spp and *Fasciolopsis buski*	17·8 (0·039 to 123·0)	3·37 (0·435 to 12·9)	6·88 (0·618 to 29·7)	0·059 (0·008 to 0·237)	15·9 (2·30 to 55·7)	26·1 (3·41 to 95·8)	29·2 (1·07 to 155·1)	6·78 (0·151 to 38·3)	0·326 (0·044 to 1·33)	0·759 (0·003 to 5·15)	2·66 (0·125 to 16·8)	0·718 (0·100 to 2·89)	7·60 (1·52 to 23·9)	0 (0 to 0)	0·298 (0·042 to 1·20)	0·675 (0·082 to 2·57)	55·5 (1·53 to 266·0)
		Minute intestinal flukes¶	0 (0 to 0)	0 (0 to 0)	0 (0 to 0)	0 (0 to 0)	0 (0 to 0)	0 (0 to 0)	2·12 (0·391 to 6·93)	2·07 (0·372 to 6·55)	0 (0 to 0)	1·17 (0·144 to 4·58)	0 (0 to 0)	0·367 (0·066 to 1·16)	0·963 (0·174 to 3·11)	1·11 (0·200 to 3·52)	0·394 (0·052 to 1·32)	2·09 (0·283 to 7·29)	2·09 (0·335 to 6·98)
		*Opisthorchis* spp‖	0 (0 to 0)	0 (0 to 0)	0 (0 to 0)	0 (0 to 0)	0 (0 to 0)	0 (0 to 0)	1·76 (0·211 to 7·05)	0·276 (0·016 to 1·40)	0·005 (<0·001 to 0·022)	0·271 (0·015 to 1·36)	0·015 (<0·001 to 0·074)	0 (0 to 0)	0 (0 to 0)	0 (0 to 0)	0 (0 to 0)	0 (0 to 0)	6·84 (0·610 to 31·7)
		*Paragonimus* spp	0·227 (<0·001 to 1·62)	2·77 (0·069 to 17·9)	0·073 (0·001 to 0·528)	0·166 (0·002 to 0·999)	0·481 (0·111 to 1·67)	0·116 (0·002 to 0·697)	0·329 (0·006 to 1·99)	0·678 (0·002 to 4·94)	0 (0 to 0)	0·026 (<0·001 to 0·135)	0 (0 to 0)	0 (0 to 0)	0 (0 to 0)	0 (0 to 0)	0·131 (0·001 to 0·929)	5·26 (1·13 to 17·1)	10·0 (0·274 to 72·5)
Bacterial toxins			0·010 (0·001 to 0·040)	0·009 (<0·001 to 0·029)	0·012 (0·001 to 0·040)	0·012 (0·006 to 0·021)	0·009 (0·003 to 0·026)	0·014 (0·003 to 0·044)	0·018 (0·004 to 0·052)	0·018 (0·004 to 0·052)	0·045 (0·023 to 0·076)	0·048 (0·019 to 0·099)	0·217 (0·044 to 0·863)	0·034 (0·003 to 0·138)	0·041 (0·008 to 0·149)	0·034 (0·003 to 0·138)	0·004 (0·002 to 0·007)	0·001 (<0·001 to 0·003)	0·007 (0·001 to 0·025)
	*Clostridium botulinum*		0·010 (0·001 to 0·040)	0·009 (<0·001 to 0·029)	0·012 (0·001 to 0·040)	0·012 (0·006 to 0·021)	0·009 (0·003 to 0·026)	0·014 (0·003 to 0·044)	0·018 (0·004 to 0·052)	0·018 (0·004 to 0·052)	0·045 (0·023 to 0·076)	0·048 (0·019 to 0·099)	0·217 (0·044 to 0·863)	0·034 (0·003 to 0·138)	0·041 (0·008 to 0·149)	0·034 (0·003 to 0·138)	0·004 (0·002 to 0·007)	0·001 (<0·001 to 0·003)	0·007 (0·001 to 0·025)
Chemical hazards			126·4 (48·3 to 270·7)	112·4 (49·1 to 227·0)	119·5 (50·0 to 235·0)	144·4 (63·2 to 313·7)	195·7 (100·7 to 414·0)	264·3 (101·7 to 659·1)	517·7 (180·8 to 1067·3)	844·0 (277·0 to 1840·9)	112·3 (47·3 to 265·8)	251·5 (84·7 to 586·3)	306·1 (120·5 to 712·9)	61·7 (24·9 to 157·0)	180·4 (76·0 to 465·8)	195·3 (60·5 to 564·9)	150·2 (83·1 to 254·0)	408·9 (126·1 to 1020·4)	405·2 (152·0 to 931·3)
	Organic pollutants		0·011 (0·002 to 0·040)	0·032 (0·005 to 0·111)	0·124 (0·011 to 0·474)	0·088 (0·012 to 0·361)	0·031 (0·007 to 0·096)	0·029 (0·005 to 0·094)	0·115 (0·005 to 0·659)	0·073 (0·008 to 0·320)	0·125 (0·039 to 0·332)	0·384 (0·063 to 1·27)	0·537 (0·056 to 2·19)	0·014 (0·002 to 0·046)	0·317 (0·023 to 1·32)	0·025 (0·003 to 0·092)	0·041 (0·006 to 0·159)	0·021 (0·004 to 0·065)	0·058 (0·010 to 0·209)
		Dioxin	0·011 (0·002 to 0·04)	0·032 (0·005 to 0·111)	0·124 (0·011 to 0·474)	0·088 (0·012 to 0·361)	0·031 (0·007 to 0·096)	0·029 (0·005 to 0·094)	0·115 (0·005 to 0·659)	0·073 (0·008 to 0·320)	0·125 (0·039 to 0·332)	0·384 (0·063 to 1·27)	0·537 (0·056 to 2·19)	0·014 (0·002 to 0·046)	0·317 (0·023 to 1·32)	0·025 (0·003 to 0·092)	0·041 (0·006 to 0·159)	0·021 (0·004 to 0·065)	0·058 (0·010 to 0·209)
	Toxins and allergens		4·73 (1·31 to 12·7)	15·2 (8·15 to 26·1)	9·46 (5·00 to 16·8)	5·28 (2·26 to 10·8)	13·5 (6·39 to 24·7)	4·10 (1·62 to 8·66)	10·2 (3·56 to 27·3)	12·6 (2·82 to 34·8)	3·91 (1·58 to 7·70)	2·67 (1·05 to 5·84)	7·99 (2·85 to 19·1)	6·08 (1·72 to 15·8)	6·02 (2·47 to 14·0)	5·23 (2·00 to 11·5)	3·56 (1·17 to 8·09)	3·57 (1·23 to 8·35)	29·6 (11·1 to 73·9)
		Aflatoxin B1	0·533 (0·041 to 2·23)	11·1 (5·64 to 19·6)	5·53 (2·43 to 11·5)	1·34 (0·246 to 4·81)	8·21 (3·30 to 17·8)	0·493 (0·083 to 1·53)	7·17 (1·48 to 24·0)	9·14 (1·02 to 30·0)	0·101 (0·065 to 0·149)	0·427 (0·127 to 1·17)	4·90 (0·793 to 16·3)	0·055 (0·011 to 0·158)	1·39 (0·563 to 2·86)	1·07 (0·167 to 3·99)	0·019 (0·005 to 0·052)	0·394 (0·157 to 0·777)	26·1 (8·41 to 69·4)
		Aflatoxin M1	0·008 (<0·001 to 0·030)	0·008 (0·002 to 0·024)	0·064 (0·014 to 0·196)	0·004 (<0·001 to 0·014)	0·005 (0·002 to 0·014)	0·003 (<0·001 to 0·009)	0·006 (0·002 to 0·017)	0·015 (0·005 to 0·032)	<0·001 (<0·001 to <0·001)	0·009 (0·003 to 0·024)	0·002 (<0·001 to 0·006)	0·002 (<0·001 to 0·007)	0·015 (0·007 to 0·029)	0·035 (0·006 to 0·112)	<0·001 (<0·001 to 0·001)	0·002 (<0·001 to 0·004)	0·003 (<0·001 to 0·019)
		Cassava cyanide	0 (0 to 0)	0·283 (0·073 to 0·777)	0·170 (0·064 to 0·381)	0 (0 to 0)	0 (0 to 0)	0 (0 to 0)	0 (0 to 0)	0 (0 to 0)	0 (0 to 0)	0 (0 to 0)	0 (0 to 0)	0 (0 to 0)	0 (0 to 0)	0 (0 to 0)	0 (0 to 0)	0 (0 to 0)	0 (0 to 0)
		Peanut allergens	4·19 (0·946 to 12·1)	3·84 (0·967 to 10·4)	3·69 (1·12 to 9·22)	3·93 (1·55 to 7·98)	5·27 (1·60 to 13·0)	3·60 (1·23 to 8·16)	3·06 (0·767 to 7·37)	3·49 (0·669 to 10·5)	3·81 (1·48 to 7·58)	2·24 (0·701 to 5·34)	3·09 (1·13 to 6·44)	6·03 (1·64 to 15·7)	4·61 (1·29 to 12·4)	4·13 (1·27 to 10·2)	3·54 (1·15 to 8·07)	3·17 (0·849 to 7·86)	3·47 (1·01 to 8·94)
	Metals		121·6 (43·3 to 264·7)	97·2 (34·1 to 212·9)	110·0 (40·0 to 225·7)	139·0 (57·5 to 307·3)	182·1 (87·8 to 401·4)	260·2 (97·7 to 654·3)	507·3 (173·2 to 1,056·4)	831·3 (263·4 to 1,827·9)	108·3 (43·3 to 262·1)	248·5 (81·6 to 583·6)	297·6 (113·2 to 703·2)	55·6 (20·2 to 151·6)	174·1 (69·3 to 458·8)	190·0 (55·3 to 558·7)	146·6 (80·2 to 250·2)	405·3 (122·8 to 1,016·6)	375·5 (124·7 to 905·9)
		Inorganic arsenic	60·7 (16·5 to 169·3)	34·8 (6·54 to 105·0)	29·7 (6·93 to 87·7)	115·7 (50·2 to 275·2)	96·1 (24·3 to 309·1)	106·1 (24·5 to 359·4)	225·2 (71·6 to 585·4)	466·2 (99·6 to 1,439·1)	41·2 (21·0 to 84·2)	126·4 (33·4 to 360·5)	172·4 (38·3 to 549·6)	21·1 (4·10 to 73·8)	72·5 (14·0 to 245·4)	59·0 (8·76 to 209·6)	81·6 (40·3 to 171·5)	220·0 (88·1 to 505·0)	206·4 (55·0 to 667·1)
		Cadmium	<0·001 (<0·001 to 0·002)	<0·001 (<0·001 to 0·001)	<0·001 (<0·001 to 0·001)	<0·001 (<0·001 to <0·001)	<0·001 (<0·001 to <0·001)	<0·001 (<0·001 to <0·001)	0·076 (0·002 to 0·417)	0·048 (<0·001 to 0·296)	<0·001 (<0·001 to 0·001)	<0·001 (<0·001 to <0·001)	<0·001 (<0·001 to <0·001)	<0·001 (<0·001 to 0·001)	<0·001 (<0·001 to 0·001)	<0·001 (<0·001 to <0·001)	0·037 (0·008 to 0·136)	0·016 (0·006 to 0·036)	0·004 (<0·001 to 0·012)
		Lead**	48·1 (−2·60 to 145·7)	38·9 (−1·62 to 116·0)	51·3 (−1·19 to 150·8)	20·2 (−0·100 to 102·4)	16·1 (−0·620 to 102·2)	29·9 (−1·26 to 192·9)	214·7 (−14·1 to 615·7)	334·9 (8·81 to 779·3)	50·9 (−2·24 to 200·2)	99·2 (−6·27 to 331·2)	75·1 (−6·52 to 192·2)	19·4 (−0·652 to 96·4)	53·4 (−0·669 to 259·1)	86·2 (0·329 to 418·5)	33·9 (−3·25 to 102·7)	178·3 (−10·5 to 778·0)	95·0 (−2·92 to 394·5)
		Methylmercury	12·9 (4·92 to 33·4)	23·5 (9·68 to 46·7)	29·0 (11·4 to 62·7)	3·14 (1·79 to 5·15)	69·9 (37·7 to 120·0)	124·2 (34·3 to 348·6)	67·3 (22·6 to 155·3)	30·2 (10·7 to 79·7)	16·1 (8·46 to 28·1)	23·0 (11·2 to 43·7)	50·2 (14·5 to 155·7)	15·1 (6·32 to 33·9)	48·2 (20·8 to 102·7)	44·9 (14·3 to 110·6)	31·1 (15·2 to 60·2)	6·96 (3·58 to 12·4)	74·1 (19·0 to 230·9)

Data are mean disability-adjusted life-years (95% uncertainty interval) per 100 000 population. For WHO subregions see the appendix (p 4). AFR=African region. AMR=region of the Amrericas. EMR=Eastern Mediterranean region. EUR=European region. SEAR=South-East Asia region. WPR=Western Pacific region.

* Includes other thermotolerant species.

† Negative lower bounds in these estimates indicate considerable uncertainty around the attributable fractions used to derive these estimates, resulting from some diarrhoeal pathogens for which asymptomatic carriage by individuals is common or data are sparse; these negative lower bounds are shown to be transparent about this uncertainty. For exposures with negative point estimates for the attributable fraction, indicating very weak confidence about the hazard causing disease (atypical enteropathogenic *Escherichia coli* [illnesses and deaths] and heat-labile toxin-producing enterotoxigenic *E coli* [deaths]), we opted not to include the associated burden.

‡ Includes diarrhoeal and invasive disease.

§ Includes acquired and congenital disease.

¶ Includes *Echinostoma* spp, *Heterophyes* spp, *Metagonimus* spp, and other foodborne intestinal trematode species with the exception of *Fasciolopsis buski*.

‖ Includes disease caused by *Opisthorchis viverrini* and *Opisthorchis felineus*.

** The negative lower bound of the uncertainty interval results from propagation of the negative lower bound of the Institute for Health Metrics and Evaluation lead burden estimate.

**Table 1:** Mean global number of foodborne illnesses, deaths, years lived with disability, years of life lost, and disability-adjusted life-years, 2021

			**Foodborne illnesses**	**Foodborne deaths**	**Foodborne years lived with disability**	**Foodborne years of life lost**	**Foodborne disability-adjusted life-years**
Total			866 000 000 (680 000 000 to 1 090 000 000)	1 520 000 (783 000 to 2 510 000)	8 360 000 (6 180 000 to 11 400 000)	48 800 000 (31 800 000 to 71 800 000)	57 100 000 (39 400 000 to 81 100 000)
Diarrhoeal disease hazards			666 000 000 (483 000 000 to 884 000 000)	296 000 (220 000 to 388 000)	628 000 (409 000 to 879 000)	16 900 000 (12 700 000 to 22 400 000)	17 600 000 (13 300 000 to 23 100 000)
	Viruses		80 200 000 (46 200 000 to 129 000 000)	40 100 (17 800 to 69 200)	69 100 (38 000 to 114 000)	2 580 000 (1 160 000 to 4 430 000)	2 650 000 (1 210 000 to 4 510 000)
		Norovirus	54 800 000 (28 000 000 to 97 100 000)	14 100 (5 330 to 28 600)	48 200 (23 900 to 83 900)	760 000 (326 000 to 1 460 000)	809 000 (356 000 to 1 540 000)
		Rotavirus	25 400 000 (7 990 000 to 50 500 000)	26 000 (6 160 to 51 700)	20 900 (6 540 to 43 500)	1 820 000 (384 000 to 3 560 000)	1 840 000 (392 000 to 3 600 000)
	Bacteria		527 000 000 (359 000 000 to 718 000 000)	225 000 (154 000 to 314 000)	516 000 (332 000 to 771 000)	12 600 000 (8 880 000 to 17 600 000)	13 100 000 (9 310 000 to 18 200 000)
		*Campylobacter jejuni* or Campylobacter coli*	148 000 000 (83 500 000 to 239 000 000)	35 400 (17 200 to 58 900)	144 000 (80 200 to 229 000)	2 000 000 (1 010 000 to 3 310 000)	2 150 000 (1 100 000 to 3 510 000)
		Enteroaggregative *Escherichia coli*†	9 100 000 (−89 300 000 to 99 200 000)	13 000 (−21 500 to 53 200)	6320 (−67 200 to 78 600)	696 000 (−1 160 000 to 2 900 000)	702 000 (−1 160 000 to 2 920 000)
		Enteropathogenic *Escherichia coli*†	21 800 000 (592 000 to 49 300 000)	8260 (−1 390 to 20 800)	16 100 (405 to 38 600)	523 000 (−93 000 to 1 310 000)	540 000 (−74 600 to 1 340 000)
		Enterotoxigenic *Escherichia coli*	131 000 000 (72 300 000 to 220 000 000)	28 900 (14 600 to 51 800)	94 100 (48 600 to 166 000)	1 700 000 (865 000 to 2 930 000)	1 790 000 (945 000 to 3 030 000)
		Shiga toxin-producing *Escherichia coli*	51 900 000 (20 700 000 to 113 000 000)	20 500 (8630 to 42 000)	53 300 (22 300 to 112 000)	1 190 000 (522 000 to 2 320 000)	1 240 000 (546 000 to 2 410 000)
		Non-typhoidal *Salmonella enterica*‡	23 500 000 (11 500 000 to 42 700 000)	55 100 (30 700 to 102 000)	21 500 (10 400 to 38 900)	3 560 000 (1 900 000 to 7 020 000)	3 580 000 (1 920 000 to 7 040 000)
		*Shigella* spp	118 000 000 (49 100 000 to 226 000 000)	42 500 (19 100 to 80 400)	103 000 (41 900 to 208 000)	2 150 000 (1 080 000 to 3 610 000)	2 250 000 (1 130 000 to 3 820 000)
		*Vibrio cholerae*	23 000 000 (5 390 000 to 67 000 000)	21 400 (5800 to 57 900)	77 700 (15 600 to 209 000)	744 000 (230 000 to 1 740 000)	821 000 (252 000 to 1 970 000)
	Protozoa		59 100 000 (28 900 000 to 103 000 000)	31 000 (15 400 to 59 600)	42 600 (19 700 to 79 300)	1 790 000 (887 000 to 3 320 000)	1 830 000 (913 000 to 3 370 000)
		*Cryptosporidium* spp	16 200 000 (6 800 000 to 28 300 000)	11 400 (4820 to 21 400)	12 100 (5070 to 22 700)	757 000 (326 000 to 1 460 000)	769 000 (332 000 to 1 480 000)
		*Cyclospora cayetanensis*†	10 200 000 (−841 000 to 31 800 000)	14 600 (3290 to 44 000)	7140 (−589 to 22 300)	850 000 (188 000 to 2 270 000)	857 000 (192 000 to 2 290 000)
		*Entamoeba histolytica*	4 660 000 (618 000 to 15 400 000)	5020 (740 to 15 600)	3180 (413 to 11 000)	180 000 (33 900 to 524 000)	183 000 (34 400 to 534 000)
		*Giardia duodenalis*	28 000 000 (8 290 000 to 64 000 000)	0 (0 to 0)	20 300 (5330 to 48 000)	0 (0 to 0)	20 300 (5330 to 48 000)
Invasive disease hazards			194 000 000 (145 000 000 to 238 000 000)	108 000 (72 900 to 157 000)	3 230 000 (2 210 000 to 5 070 000)	6 600 000 (4 170 000 to 10 100 000)	9 830 000 (6 880 000 to 14 000 000)
	Viruses		20 000 000 (13 100 000 to 26 600 000)	11 900 (4860 to 22 900)	59 300 (31 100 to 98 800)	761 000 (291 000 to 1 470 000)	821 000 (334 000 to 1 530 000)
		Hepatitis A virus	20 000 000 (13 100 000 to 26 600 000)	11 900 (4860 to 22 900)	59 300 (31 100 to 98 800)	761 000 (291 000 to 1 470 000)	821 000 (334 000 to 1 530 000)
	Bacteria		3 780 000 (1 970 000 to 6 160 000)	62 500 (32 100 to 107 000)	100 000 (57 100 to 167 000)	4 030 000 (1 900 000 to 7 300 000)	4 130 000 (1 960 000 to 7 430 000)
		*Brucella* spp	119 000 (51 800 to 312 000)	11 (2 to 37)	5160 (1940 to 14 400)	577 (109 to 1920)	5730 (2180 to 16 600)
		*Listeria monocytogenes*	22 800 (8480 to 71 800)	5280 (1910 to 16 300)	4230 (1350 to 13 500)	188 000 (68 300 to 576 000)	192 000 (69 700 to 588 000)
		*Mycobacterium bovis, Mycobacterium caprae*, or *Mycobacterium orygis*	138 000 (69 900 to 256 000)	15 800 (8280 to 28 600)	61 800 (27 200 to 123 000)	627 000 (337 000 to 1 100 000)	689 000 (367 000 to 1 220 000)
		*Salmonella enterica* serotype Paratyphi A, B, and C	757 000 (296 000 to 1 400 000)	5340 (1740 to 11 700)	2100 (753 to 4290)	397 000 (133 000 to 878 000)	400 000 (134 000 to 880 000)
		*Salmonella enterica* serotype Typhi	2 740 000 (1 060 000 to 4 940 000)	36 000 (11 900 to 74 600)	26 700 (9430 to 51 200)	2 810 000 (944 000 to 5 860 000)	2 840 000 (960 000 to 5 910 000)
	Parasites		171 000 000 (122 000 000 to 214 000 000)	33 800 (24 100 to 50 900)	3 070 000 (2 060 000 to 4 900 000)	1 820 000 (1 290 000 to 2 730 000)	4 890 000 (3 490 000 to 7 100 000)
		*Toxoplasma gondii*§	26 100 000 (19 800 000 to 31 800 000)	495 (239 to 880)	841 000 (507 000 to 1 320 000)	45 700 (22 100 to 81 300)	887 000 (545 000 to 1 380 000)
		*Trypanosoma cruzi*	74 300 (24 400 to 135 000)	5740 (5010 to 6540)	246 000 (77 900 to 485 000)	276 000 (241 000 to 315 000)	522 000 (349 000 to 768 000)
		*Echinococcus granulosus*	261 000 (124 000 to 497 000)	1610 (495 to 3950)	34 400 (11 900 to 81 900)	93 500 (28 700 to 229 000)	128 000 (45 900 to 293 000)
		*Echinococcus multilocularis*	989 (106 to 4110)	289 (9 to 1410)	1330 (89 to 6920)	7960 (253 to 38 800)	9290 (376 to 43 900)
		*Taenia solium*	247 000 (156 000 to 358 000)	13 300 (9980 to 17 300)	589 000 (336 000 to 924 000)	708 000 (529 000 to 924 000)	1 300 000 (903 000 to 1 800 000)
		*Ascaris* spp	143 000 000 (95 000 000 to 185 000 000)	1160 (535 to 2070)	186 000 (120 000 to 246 000)	93 100 (42 800 to 166 000)	279 000 (174 000 to 394 000)
		*Trichinella* spp	1540 (699 to 3550)	4 (0 to 23)	117 (47 to 288)	218 (27 to 1330)	334 (86 to 1510)
		*Clonorchis sinensis*	56 200 (13 800 to 149 000)	10 200 (2480 to 27 300)	375 000 (76 700 to 1 060 000)	546 000 (132 000 to 1 460 000)	921 000 (215 000 to 2 550 000)
		*Fasciola* spp and *Fasciolopsis buski*	133 000 (27 700 to 425 000)	0 (0 to 0)	588 000 (111 000 to 2 010 000)	0 (0 to 0)	588 000 (111 000 to 2 010 000)
		Minute intestinal flukes¶	1 060 000 (189 000 to 3 310 000)	0 (0 to 0)	90 200 (15 700 to 289 000)	0 (0 to 0)	90 200 (15 700 to 289 000)
		*Opisthorchis* spp‖	15 400 (2740 to 54 000)	36 (4 to 163)	27 200 (3330 to 115 000)	1610 (176 to 7280)	28 800 (3540 to 123 000)
		*Paragonimus* spp	18 700 (803 to 107 000)	962 (286 to 2060)	92 400 (3950 to 552 000)	43 000 (12 800 to 92 300)	135 000 (22 900 to 597 000)
Bacterial toxins			667 (375 to 1250)	35 (14 to 74)	17 (5 to 43)	1510 (602 to 3220)	1530 (611 to 3240)
	*Clostridium botulinum*		667 (375 to 1250)	35 (14 to 74)	17 (5 to 43)	1510 (602 to 3220)	1530 (611 to 3240)
Chemical hazards			6 260 000 (3 360 000 to 10 300 000)	1 120 000 (404 000 to 2 100 000)	4 500 000 (2 790 000 to 6 870 000)	25 300 000 (9 450 000 to 47 800 000)	29 800 000 (12 900 000 to 53 100 000)
	Organic pollutants		37 000 (16 900 to 78 900)	0 (0 to 0)	7730 (2340 to 18 500)	0 (0 to 0)	7730 (2340 to 18 500)
		Dioxin	37 000 (16 900 to 78 900)	0 (0 to 0)	7730 (2340 to 18 500)	0 (0 to 0)	7730 (2340 to 18 500)
	Toxins and allergens		295 000 (158 000 to 524 000)	13 200 (5660 to 26 800)	292 000 (113 000 to 578 000)	414 000 (182 000 to 837 000)	706 000 (394 000 to 1 210 000)
		Aflatoxin B1	14 000 (5920 to 28 000)	13 200 (5570 to 26 400)	2070 (860 to 4270)	410 000 (178 000 to 809 000)	412 000 (179 000 to 813 000)
		Aflatoxin M1	29 (16 to 48)	27 (15 to 45)	5 (3 to 8)	890 (484 to 1570)	895 (487 to 1570)
		Cassava cyanide	145 (50 to 335)	31 (10 to 72)	358 (119 to 837)	2170 (719 to 5070)	2530 (837 to 5890)
		Peanut allergens	281 000 (143 000 to 512 000)	5 (0 to 16)	290 000 (111 000 to 576 000)	269 (18 to 927)	290 000 (111 000 to 577 000)
	Metals		5 920 000 (3 020 000 to 9 940 000)	1 110 000 (388 000 to 2 080 000)	4 200 000 (2 510 000 to 6 540 000)	24 800 000 (8 950 000 to 47 400 000)	29 000 000 (12 100 000 to 52 300 000)
		Inorganic arsenic	2 210 000 (789 000 to 5 440 000)	641 000 (271 000 to 1 390 000)	270 000 (117 000 to 571 000)	15 100 000 (6 250 000 to 33 900 000)	15 400 000 (6 370 000 to 34 500 000)
		Cadmium	202 (31 to 936)	47 (5 to 294)	304 (56 to 1240)	1100 (120 to 5670)	1410 (208 to 7340)
		Lead**	1 700 000 (−68 200 to 4 100 000)	466 000 (−52 300 to 1 180 000)	1 500 000 (458 000 to 2 940 000)	9 700 000 (−1 090 000 to 24 100 000)	11 200 000 (−149 000 to 26 500 000)
		Methylmercury	2 020 000 (1 190 000 to 3 620 000)	0 (0 to 0)	2 430 000 (1 320 000 to 4 380 000)	0 (0 to 0)	2 430 000 (1 320 000 to 4 380 000)

Data in parentheses are 95% uncertainty intervals.

* Includes other thermotolerant species.

† Negative lower bounds in these estimates indicate considerable uncertainty around the attributable fractions used to derive these estimates, resulting from some diarrhoeal pathogens for which asymptomatic carriage by individuals is common or data are sparse; these negative lower bounds are shown to be transparent about this uncertainty. For exposures with negative point estimates for the attributable fraction, indicating very weak confidence about the hazard causing disease (atypical enteropathogenic *Escherichia coli* [illnesses and deaths] and heat-labile toxin-producing enterotoxigenic *E coli* [deaths]), we opted not to include the associated burden.

‡ Includes diarrhoeal and invasive disease.

§ Includes acquired and congenital disease.

¶ Includes *Echinostoma* spp, *Heterophyes* spp, *Metagonimus* spp, and other foodborne intestinal trematode species with the exception of *Fasciolopsis buski*.

‖ Includes disease caused by *Opisthorchis viverrini* and *Opisthorchis felineus*.

** The negative lower bound of the uncertainty interval results from propagation of the negative lower bound of the Institute for Health Metrics and Evaluation lead burden estimate.

The foodborne chemical hazards caused 1·12 million (95% UI 0·404–2·10) deaths.^9^ Despite chemical hazards only resulting in 0·7% of all foodborne illnesses globally, they were responsible for 73% of all deaths due to contaminated food. Inorganic arsenic was responsible for 42% and lead for 31% of all deaths due to contaminated food. The burden from inorganic arsenic exposure was largely due to ischaemic heart disease, whereas the burden from lead results from cardiovascular diseases, primarily ischaemic heart disease and both ischaemic and haemorrhagic strokes.^9^

**Figure 1:**
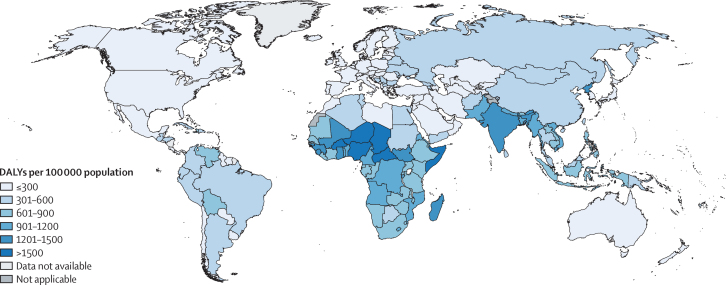
Foodborne DALYs per 100 000 population by country, 2021 DALYs=disability-adjusted life-years.

The highest mean rates of foodborne illness per 100 000 population were estimated to have occurred in the African region C, African region D, and South-East Asia region CD clusters of the 17 subregional clusters, whereas the lowest estimated rates were in cluster A countries of all regions (table 2). The highest rates of population burden for individual countries occurred in parts of sub-Saharan Africa and the Horn of Africa (figure 1).

The highest rates of foodborne DALYs occurred in the African region subregions C and D, where diarrhoeal disease hazards made the greatest contribution to foodborne disease burden, and in the South-East Asia region CD subregion, where chemical hazards were the greatest contributors to overall burden (appendix p 6).

The incidence of illness in children younger than 5 years was 2·7 times higher than in those aged 5 years or older, and the DALY rate was 4·3 times higher (table 3). The highest rates of foodborne burden of disease in children younger than 5 years were due to methylmercury, non-typhoidal *Salmonella enterica*, and rotavirus. By contrast, in individuals aged 5 years or older, the greatest burden resulted from inorganic arsenic, lead, and non-typhoidal *S enterica*.

**Table 3:** Mean rates of foodborne illnesses, deaths, and DALYs per 100 000 population globally by age group, 2021

			**Age <5 years**			**Age ≥5 years**		
			Illnesses	Deaths	DALYs	Illnesses	Deaths	DALYs
Total			26 077·0 (20 444·4 to 31 183·3)	21·2 (15·9 to 27·7)	2450·6 (1947·9 to 3105·4)	9582·9 (7247·3 to 12 410·6)	19·2 (8·96 to 32·9)	563·5 (327·3 to 888·6)
Diarrhoeal disease hazards			10 718·8 (7828·5 to 13 847·2)	17·5 (13·0 to 23·3)	1615·9 (1201·1 to 2138·6)	8240·8 (5875·8 to 11 114·9)	2·47 (1·72 to 3·41)	92·1 (67·3 to 126·0)
	Viruses		2089·3 (1280·1 to 3018·3)	3·31 (1·39 to 5·88)	305·1 (130·2 to 540·8)	917·7 (504·6 to 1542·2)	0·247 (0·105 to 0·492)	8·13 (3·77 to 14·7)
		Norovirus	1128·5 (661·1 to 1848·8)	0·824 (0·349 to 1·51)	77·1 (33·1 to 140·2)	654·9 (322·3 to 1194·4)	0·118 (0·037 to 0·263)	3·99 (1·58 to 8·09)
		Rotavirus	960·8 (393·8 to 1700·7)	2·48 (0·501 to 4·94)	228·0 (46·1 to 453·2)	262·8 (71·5 to 552·0)	0·128 (0·031 to 0·308)	4·14 (1·02 to 9·13)
	Bacteria		7727·8 (5157·3 to 10 497·5)	12·3 (8·65 to 17·1)	1131·0 (800·5 to 1572·4)	6587·5 (4446·7 to 9118·0)	1·97 (1·32 to 2·89)	75·4 (52·1 to 108·0)
		*Campylobacter jejuni* or Campylobacter coli*	2748·1 (1724·6 to 4040·4)	2·27 (1·18 to 3·73)	212·4 (110·9 to 346·9)	1801·5 (979·8 to 2992·2)	0·279 (0·130 to 0·501)	9·84 (4·85 to 16·6)
		Enteroaggregative *Escherichia coli*†	148·4 (−1420·0 to 1714·6)	0·755 (−1·32 to 3·13)	69·2 (−120·0 to 286·5)	112·4 (−1120·0 to 1221·9)	0·109 (−0·180 to 0·454)	3·25 (−5·05 to 13·4)
		Enteropathogenic *Escherichia coli*†	429·0 (11·5 to 953·1)	0·623 (−0·111 to 1·58)	57·5 (−9·78 to 145·5)	262·2 (7·09 to 603·4)	0·056 (−0·009 to 0·151)	2·09 (−0·137 to 5·05)
		Enterotoxigenic *Escherichia coli*	1876·0 (1086·5 to 2912·1)	1·86 (0·904 to 3·32)	172·2 (84·5 to 306·0)	1644·5 (883·5 to 2774·8)	0·226 (0·109 to 0·418)	8·72 (4·57 to 15·1)
		Shiga toxin-producing *Escherichia coli*	721·6 (334·4 to 1439·0)	1·07 (0·445 to 2·20)	99·6 (41·4 to 203·4)	652·8 (256·9 to 1432·2)	0·184 (0·069 to 0·417)	7·92 (3·16 to 17·5)
		Non-typhoidal *Salmonella enterica*‡	390·8 (225·6 to 626·9)	3·20 (1·69 to 6·05)	291·3 (154·4 to 548·8)	290·3 (137·5 to 542·8)	0·464 (0·251 to 0·877)	22·4 (11·5 to 46·9)
		*Shigella* spp	1333·3 (698·8 to 2440·2)	2·11 (1·07 to 3·62)	194·7 (99·2 to 332·7)	1511·6 (626·5 to 2937·9)	0·393 (0·137 to 0·791)	13·0 (5·36 to 24·8)
		*Vibrio cholerae*	80·6 (23·8 to 201·1)	0·371 (0·135 to 0·769)	34·2 (12·4 to 71·5)	312·3 (72·5 to 912·3)	0·262 (0·061 to 0·730)	8·20 (2·22 to 20·8)
	Protozoa		901·7 (494·7 to 1459·4)	1·96 (0·920 to 3·78)	179·8 (84·8 to 346·2)	735·7 (347·1 to 1 291·1)	0·247 (0·105 to 0·504)	8·51 (3·92 to 16·9)
		*Cryptosporidium* spp	337·8 (155·3 to 614·5)	0·957 (0·398 to 1·97)	87·8 (36·6 to 180·7)	193·6 (78·5 to 348·4)	0·068 (0·027 to 0·139)	2·43 (1·08 to 4·64)
		*Cyclospora cayetanensis*†	101·0 (−8·07 to 309·5)	0·892 (0·176 to 2·84)	81·6 (16·1 to 259·8)	132·6 (−10·3 to 423·1)	0·119 (0·026 to 0·358)	4·24 (0·931 to 12·9)
		*Entamoeba histolytica*	26·5 (4·10 to 80·5)	0·107 (0·025 to 0·268)	9·80 (2·29 to 24·6)	62·3 (8·15 to 204·3)	0·060 (0·007 to 0·197)	1·62 (0·224 to 4·92)
		*Giardia duodenalis*	436·4 (165·4 to 906·7)	0 (0 to 0)	0·561 (0·189 to 1·27)	347·3 (98·6 to 812·8)	0 (0 to 0)	0·229 (0·058 to 0·567)
Invasive disease hazards			15 016·7 (10 299·6 to 19 131·3)	3·62 (1·84 to 6·52)	418·0 (247·7 to 686·4)	1287·3 (1024·6 to 1 536·2)	1·16 (0·820 to 1·64)	97·3 (69·6 to 137·2)
	Viruses		177·9 (105·4 to 260·2)	0·530 (0·168 to 1·12)	48·8 (15·8 to 102·8)	260·8 (168·8 to 351·0)	0·116 (0·051 to 0·225)	6·81 (3·08 to 12·5)
		Hepatitis A virus	177·9 (105·4 to 260·2)	0·530 (0·168 to 1·12)	48·8 (15·8 to 102·8)	260·8 (168·8 to 351·0)	0·116 (0·051 to 0·225)	6·81 (3·08 to 12·5)
	Bacteria		170·3 (72·8 to 297·5)	2·72 (1·06 to 5·43)	247·4 (97·2 to 493·3)	36·5 (19·4 to 59·4)	0·613 (0·337 to 1·03)	34·1 (17·5 to 59·7)
		*Brucella* spp	0·352 (0·153 to 0·922)	<0·001 (<0·001 to <0·001)	0·018 (0·007 to 0·053)	1·62 (0·705 to 4·25)	<0·001 (<0·001 to <0·001)	0·078 (0·030 to 0·225)
		*Listeria monocytogenes*	0·315 (0·086 to 1·05)	0·063 (0·020 to 0·194)	5·94 (1·86 to 18·3)	0·287 (0·110 to 0·898)	0·067 (0·024 to 0·208)	2·11 (0·755 to 6·48)
		*Mycobacterium bovis, Mycobacterium caprae,* or *Mycobacterium orygis*	0·981 (0·585 to 1·49)	0·133 (0·077 to 0·210)	12·5 (7·24 to 19·7)	1·82 (0·914 to 3·41)	0·207 (0·107 to 0·381)	8·40 (4·36 to 15·2)
		*Salmonella enterica* serotype Paratyphi A, B, and C	28·3 (10·7 to 53·7)	0·262 (0·081 to 0·597)	23·7 (7·35 to 54·0)	7·85 (2·89 to 14·7)	0·050 (0·016 to 0·112)	3·32 (1·10 to 7·39)
		*Salmonella enterica* serotype Typhi	140·4 (50·1 to 264·2)	2·26 (0·724 to 4·75)	205·3 (65·8 to 431·5)	24·9 (9·67 to 43·8)	0·288 (0·096 to 0·599)	20·2 (6·79 to 41·9)
	Parasites		14 668·5 (9970·5 to 18 825·1)	0·378 (0·282 to 0·504)	121·7 (93·5 to 159·3)	989·9 (750·7 to 1 209·0)	0·434 (0·302 to 0·668)	56·4 (38·6 to 86·4)
		*Toxoplasma gondii*§	820·6 (621·6 to 1 000·4)	0·073 (0·035 to 0·130)	58·3 (37·4 to 87·8)	285·7 (216·4 to 348·3)	0 (0 to 0)	6·84 (4·02 to 11·3)
		*Trypanosoma cruzi*	0·671 (0·206 to 1·29)	0 (0 to 0)	0·014 (0·004 to 0·031)	0·969 (0·316 to 1·77)	0·080 (0·070 to 0·091)	7·26 (4·84 to 10·7)
		*Echinococcus granulosus*	1·78 (0·849 to 3·39)	0·011 (0·003 to 0·027)	1·23 (0·415 to 2·85)	3·46 (1·65 to 6·59)	0·021 (0·007 to 0·052)	1·66 (0·597 to 3·81)
		*Echinococcus multilocularis*	0 (0 to 0)	0 (0 to 0)	0 (0 to 0)	0·014 (0·001 to 0·057)	0·004 (<0·001 to 0·020)	0·129 (0·005 to 0·610)
		*Taenia solium*	5·60 (3·19 to 8·77)	0·170 (0·126 to 0·215)	23·1 (16·7 to 30·9)	2·91 (1·82 to 4·20)	0·168 (0·126 to 0·220)	15·9 (11·0 to 22·1)
		*Ascaris* spp	13 838·1 (9221·4 to 17 968·5)	0·113 (0·052 to 0·201)	28·3 (17·6 to 40·2)	679·2 (452·6 to 881·9)	0·006 (0·003 to 0·010)	1·22 (0·776 to 1·70)
		*Trichinella* spp	<0·001 (<0·001 to <0·001)	<0·001 (<0·001 to <0·001)	<0·001 (<0·001 to <0·001)	0·021 (0·010 to 0·049)	<0·001 (<0·001 to <0·001)	0·005 (0·001 to 0·021)
		*Clonorchis sinensis*	1·09 (0·267 to 2·90)	0·011 (0·003 to 0·029)	10·40 (2·19 to 29·0)	0·678 (0·166 to 1·80)	0·141 (0·034 to 0·376)	11·8 (2·75 to 32·6)
		*Fasciola* spp and *Fasciolopsis buski*	0·019 (0·004 to 0·060)	0 (0 to 0)	0·144 (0·027 to 0·494)	1·84 (0·384 to 5·90)	0 (0 to 0)	8·15 (1·54 to 27·9)
		Minute intestinal flukes¶	0·600 (0·107 to 1·87)	0 (0 to 0)	0·051 (0·009 to 0·164)	14·7 (2·61 to 45·9)	0 (0 to 0)	1·25 (0·218 to 4·00)
		*Opisthorchis* spp‖	0·009 (0·002 to 0·030)	<0·001 (<0·001 to <0·001)	0·037 (0·004 to 0·161)	0·213 (0·038 to 0·747)	<0·001 (<0·001 to 0·002)	0·397 (0·049 to 1·70)
		*Paragonimus* spp	0·011 (<0·001 to 0·060)	<0·001 (<0·001 to 0·001)	0·176 (0·028 to 0·802)	0·259 (0·011 to 1·48)	0·013 (0·004 to 0·029)	1·86 (0·316 to 8·22)
Bacterial toxins			0 (0 to 0)	0 (0 to 0)	0 (0 to 0)	0·009 (0·005 to 0·017)	<0·001 (<0·001 to 0·001)	0·021 (0·008 to 0·045)
	Clostridium botulinum**		0 (0 to 0)	0 (0 to 0)	0 (0 to 0)	0·009 (0·005 to 0·017)	<0·001 (<0·001 to 0·001)	0·021 (0·008 to 0·045)
Chemical hazards			341·5 (213·3 to 577·8)	<0·001 (<0·001 to <0·001)	416·7 (246·4 to 725·7)	54·8 (18·9 to 108·3)	15·6 (5·62 to 29·1)	374·1 (142·2 to 700·5)
	Organic pollutants		0 (0 to 0)	0 (0 to 0)	0 (0 to 0)	0·514 (0·234 to 1·10)	0 (0 to 0)	0·107 (0·032 to 0·257)
		Dioxin	0 (0 to 0)	0 (0 to 0)	0 (0 to 0)	0·514 (0·234 to 1·10)	0 (0 to 0)	0·107 (0·032 to 0·257)
	Toxins and allergens		41·5 (21·1 to 75·7)	<0·001 (<0·001 to <0·001)	42·8 (16·5 to 85·2)	0·197 (0·085 to 0·396)	0·184 (0·078 to 0·373)	5·78 (2·55 to 11·7)
		Aflatoxin B1	0 (0 to 0)	0 (0 to 0)	0 (0 to 0)	0·194 (0·082 to 0·389)	0·183 (0·077 to 0·366)	5·73 (2·49 to 11·3)
		Aflatoxin M1	0 (0 to 0)	0 (0 to 0)	0 (0 to 0)	<0·001 (<0·001 to <0·001)	<0·001 (<0·001 to <0·001)	0·012 (0·007 to 0·022)
		Cassava cyanide	<0·001 (<0·001 to 0·002)	<0·001 (<0·001 to <0·001)	0·031 (0·010 to 0·071)	0·002 (<0·001 to 0·004)	<0·001 (<0·001 to <0·001)	0·032 (0·011 to 0·075)
		Peanut allergens	41·5 (21·1 to 75·7)	<0·001 (<0·001 to <0·001)	42·8 (16·4 to 85·2)	0 (0 to 0)	<0·001 (<0·001 to <0·001)	0·004 (<0·001 to 0·013)
	Metals		300·0 (176·8 to 537·6)	0 (0 to 0)	373·9 (208·7 to 671·1)	54·1 (18·2 to 107·5)	15·4 (5·39 to 28·9)	368·2 (135·8 to 695·0)
		Inorganic arsenic	0 (0 to 0)	0 (0 to 0)	0 (0 to 0)	30·7 (11·0 to 75·5)	8·90 (3·76 to 19·3)	214·1 (88·5 to 478·6)
		Cadmium	0 (0 to 0)	0 (0 to 0)	0 (0 to 0)	0·003 (<0·001 to 0·013)	<0·001 (<0·001 to 0·004)	0·020 (0·003 to 0·102)
		Lead††	1·72 (0·459 to 3·62)	0 (0 to 0)	15·1 (4·02 to 31·7)	23·4 (−1·13 to 56·7)	6·47 (−0·727 to 16·4)	154·1 (−3·56 to 366·7)
		Methylmercury	298·3 (175·7 to 535·5)	0 (0 to 0)	358·8 (194·8 to 647·6)	0 (0 to 0)	0 (0 to 0)	0 (0 to 0)

Data in parentheses are 95% uncertainty intervals.

* Includes other thermotolerant species.

† Negative lower bounds in these estimates indicate considerable uncertainty around the attributable fractions used to derive these estimates, resulting from some diarrhoeal pathogens for which asymptomatic carriage by individuals is common or data are sparse; these negative lower bounds are shown to be transparent about this uncertainty. For exposures with negative point estimates for the attributable fraction, indicating very weak confidence about the hazard causing disease (atypical enteropathogenic *Escherichia coli* [illnesses and deaths] and heat-labile toxin-producing enterotoxigenic *E coli* [deaths]), we opted not to include the associated burden.

‡ Includes diarrhoeal and invasive disease.

§ Includes acquired and congenital disease.

¶ Includes *Echinostoma* spp, *Heterophyes* spp, *Metagonimus* spp, and other foodborne intestinal trematode species with the exception of *Fasciolopsis buski*.

‖ Includes disease caused by *Opisthorchis viverrini* and *Opisthorchis felineus*.

** Not estimated in age group <5 years.

†† The negative lower bound of the uncertainty interval results from propagation of the negative lower bound of the Institute for Health Metrics and Evaluation lead burden estimate.

**Figure 2:**
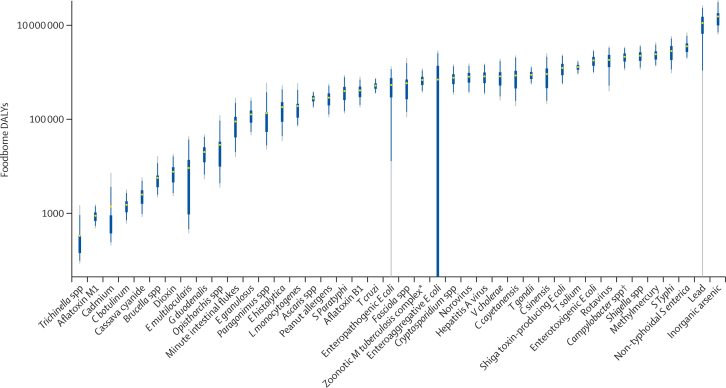
Ranking of foodborne hazards globally by DALYs, 2021 The y-axis is on a logarithmic scale. White dots indicate the mean burden, black boxes the IQR (50% UI), and black lines the 5th and 95th percentiles (90% UI; grey lines the 2·5th and 97·5th percentiles [95% UI]). *C botulinum*=*Clostridium botulinum*. *C cayetanensis=Cyclospora cayetanensis*. *C sinensis*=*Clonorchis sinensis*. DALYs=disability-adjusted life-years. *E coli*=*Escherichia coli. E granulosus*=*Echinococcus granulosus*. *E histolytica*=*Entamoeba histolytica*. *E multilocularis*=*Echinococcus multilocularis*. *G duodenalis*=*Giardia duodenalis*. *M tuberculosis*=*Mycobacterium tuberculosis*. *S enterica*=*Salmonella enterica*. *T gondii*=*Toxoplasma gondii*. *T solium*=*Taenia solium*. UI=uncertainty interval. *V cholerae*=*Vibrio cholerae*. *Includes *M bovis*, *M caprae*, or *M orygis*. †Include *Campylobacter jejuni* and *Campylobacter coli* (and other thermotolerant species).

When the hazards were ranked by global foodborne DALY estimates for 2021, the burden from inorganic arsenic and lead exposure was markedly higher than for all other hazards (figure 2). In our estimates, ischaemic heart disease contributes more than 80% of the DALYs for foodborne inorganic arsenic exposure, the majority of which result from exposure in India and China. The microbial hazard with the greatest burden was non-typhoidal *S enterica,* which included burden from both diarrhoeal and invasive illness.

The trend lines for total foodborne disease YLLs, YLDs, and DALYs for all hazards from 2000 to 2021 show a decline in burden of that time period (appendix pp 7–12). Comparing reductions in YLDs and YLLs, YLLs seemed to decline at a greater rate in Africa than YLDs.

For 2021, the estimated productivity loss was US$310 billion in nominal terms for all countries combined when all age groups were considered (appendix p 14), and US$647 billion after adjusting for purchasing power parity. High-income countries accounted for just under US$108 billion and low-income and middle-income countries US$202 billion. This loss was 0·52% of global gross domestic product (GDP), but 1·16% of GDP in low-income countries. After excluding children younger than 5 years, global productivity loss decreased from 0·52% to 0·31% of GDP (appendix p 4). Regional and global estimates mask substantial variation in national economic impacts (appendix p 14).

## Discussion

We estimated that, in 2021, transmission of the 42 hazards via contaminated food resulted in 57 million DALYs. This foodborne burden is of the same magnitude as WHO 2021 DALY estimates for tuberculosis (60 million), HIV (40 million), and malaria (52 million).^26^ Along with the estimate of lost productivity in terms of GDP, this finding reinforces the importance of food safety as a public health and economic issue. It is imperative that countries implement strategies to improve food safety to reduce this substantial burden of disease arising from contaminated food.

Not all hazards showed significant temporal trends during 2000–21, and data for several hazards were incomplete across the study period. Although the trend must be interpreted cautiously, the reduction in total foodborne DALY burden during 2000–21 is encouraging and is most apparent for reduced burden in the African region. There are several potential reasons for this decline. Improvements in food safety systems, along with water, sanitation, and hygiene are likely to play a part. Greater access to, and higher quality of medical care, diagnostic improvements and greater data availability, might also be factors.

National-level estimates reveal substantial inequalities in the global burden of foodborne diseases. Foodborne disease burden is likely to be related to socioeconomic status, as well as cultural and epidemiological mechanisms. Although the incidence of illness from some non-diarrhoeal parasitic hazards is minor in a global context, for specific localised populations they can present a high burden. Examples are foodborne transmission of *Clonorchis sinensis* in the Western Pacific region and *T cruzi* in the region of the Americas; various factors are of importance, including environmental factors that might cause localised distribution of vectors or intermediate snail hosts in specific settings.

The selection of hazards that we added to these updated estimates of the global burden of foodborne disease particularly aimed to expand the range of chemical and parasitic hazards, following feedback on the 2010 estimates, and considering emerging hazards. Some of these additional hazards, including inorganic arsenic, lead, rotavirus, and *T cruzi,* add considerably to the burden.

Estimates of the global burden from dietary exposure to inorganic arsenic, lead, methylmercury, and cadmium in food were published in 2019.^5^ The results for methylmercury and cadmium were of the same order as the estimates in this report, but the burden from inorganic arsenic and lead exposure is much higher in this report owing to the inclusion of cardiovascular diseases as health outcomes. The importance of heart disease as a major contributor to the burden from lead and inorganic arsenic exposure is consistent with other estimates.^27^, ^28^

The causal link between arsenic exposure and ischaemic heart disease has been recently demonstrated,^29^ and the resulting disease burden is described in a previous paper.^30^ About 90% of the burden from ischaemic heart disease results from mortality,^31^ and ischaemic heart disease is the second leading cause of global DALYs in 2021, after COVID-19.^17^ The high mortality, and inclusion of even a small proportion of the global burden from ischaemic heart disease, results in a large burden in the food safety context. Mortality from foodborne exposure to inorganic arsenic and lead largely accounts for the increase in mortality from the previous WHO estimate for 2010 of 420 000 deaths.^4^

Despite these 2021 estimates representing an update from the 2010 estimates published in 2015, we do not advise comparing the two due to differences in global populations, modelling approaches, and parameter inputs. Nevertheless, in the hazard ranking for infectious pathogens for both the 2010 and 2021 estimates, non-typhoidal *S enterica* (diarrhoeal and invasive illness) represents the hazard contributing the greatest burden. Even though their impact as a cause of diarrhoea has decreased due to updated attribution methods, non-typhoidal *S enterica* continues to cause a substantial burden from invasive illness.^7^

Productivity loss estimates are best considered in relation to other major economic indicators. Although estimates of total productivity losses are often lower among low-income countries, those losses tend to be higher in proportion to national GDP. Health economists tend to exclude coverage of children when estimating productivity loss as their time use prepares them for future contributions rather than directly affecting current productivity. National impacts are more relevant to national decision making. Future work will explore the influence of more comprehensive measures of health impact, the role of children, and national variation in impact.

These estimates are subject to several important limitations. Despite progress in data availability, there remain considerable data gaps in disease incidence and mortality across the world, along with uncertainties in attributing disease to specific modes of transmission. Adjustment of surveillance data to account for under-reporting and underascertainment is often based on limited country-specific data. Although the number of hazards covered has increased, many potentially foodborne hazards and health states have still not been included, such as astrovirus and *Toxocara canis*. For these reasons, values presented in this report underestimate the true burden. Economic estimates presented only include lost productivity, a very substantial underestimate of the economic impact of foodborne disease.

Available data for many of the estimates were limited to published sources, which might be biased. Disability weights and disease models can be proxies when exact matches are unavailable. The necessity of a variety of approaches to the estimation of burden from dietary exposure to chemical hazards might have biased output.

The COVID-19 pandemic caused changes to the reported incidence of many potentially foodborne diseases in some countries, particularly during 2020–21. These changes have not been well documented and have therefore not been explicitly included in our modelling. The effects of antimicrobial resistance on the burden were not addressed in our estimates.

The first estimates of the global burden of foodborne disease provided global and regional estimates that allowed countries and the global food safety community to identify priority hazards in different regions but did not reveal subregional and national variations that are important for context-specific prevention efforts. These updated estimates provide subregional and national estimates of the incidence, mortality, burden, and sources of important foodborne diseases. The expanded range of hazards compared with the previous estimates provides a more complete picture of the foodborne disease burden. The ability to examine time trends extracts greater value from the data to support decision making.

These estimates allow better targeting of foodborne disease interventions, and prioritisation of research needs. The WHO global strategy for food safety 2022–30 presents several strategic priorities for improvement, especially strengthening national food control systems.^1^

We observed a decline in the total global burden of foodborne disease over the period 2000–21. Despite this, the total burden in 2021 is still significant and preventable by systemic improvements in food safety along the food supply chain.

Although the estimated burden from inorganic arsenic and lead are substantially higher than for other hazards, it must not divert efforts to address microbial hazards. Caution must be applied when communicating these results, as dietary exposure to these metals only contribute to a small proportion of the global burden of cardiovascular diseases.

There are many tools already available to reduce the risk from foodborne microbial and parasitic hazards, such as cooking, pasteurisation, specific food preparation practices, improved irrigation practices, slaughter and meat inspection, and vaccination of animals and humans. For chemical hazards, food contamination might result from environmental patterns such as the geological nature of the soil where crops are grown, from anthropogenic activities such as mining, storage conditions of crops, and the chain of custody for allergens. Elimination of the chemical from food after contamination is unlikely. Instead, efforts must be made to prevent contamination of food at source, or by managing the ingestion of the highest food contributors. This will require engagement with stakeholders across the entire food chain, including culturally appropriate dietary advice to consumers that balances risks and benefits of different food sources.

Our intention is that these data should be used for policy development at national and international levels.^2^ Our national burden estimates will be subject to the limitations discussed here. It is to be hoped that countries without existing national burden estimates will be encouraged to improve internal foodborne disease surveillance systems and local estimates.

## FERG members

## Contributors

## Data sharing

Data collected by WHO for this study and data sources are publicly available at https://www.who.int/teams/nutrition-and-food-safety/monitoring-nutritional-status-and-food-safety-and-events/foodborne-disease-estimates/2026-edition. The analysis code is available at https://github.com/fbdburden.

## Declaration of interests

SEM reports grants and contracts from the Canadian Institutes for Health Research, National Science and Engineering Research Council, Gates Foundation, and the UK's Department for International Development, and consulting fees from Western University (London, ON, Canada). ESW reports grants and contracts from the US Centers for Disease Control and Prevention, US Department of Agriculture, Colorado Department of Public Health and Environment, Human Resources and Services Administration, National Network of Public Health Institutes, and the Council for State and Territorial Epidemiologists. BD, CdB, LV, and SMP report grants and contracts from the European Food Safety Agency. RJL reports grants and contracts from the New Zealand Institute of Public Health and Forensic Science and participation on data safety monitoring boards or advisory boards for New Zealand Food Safety. LJR reports grants and contracts from the Norwegian Research Council, Nestle (scientific services only), and Water Providers in Norway; participation on data safety monitoring boards or advisory boards for the Norwegian Scientific Committee of Food Safety and the Environment; and leadership or fiduciary role in other board, society, committee, or advocacy group, paid or unpaid for the Food and Agriculture Organization of the UN and the European Federation of Parasitologists. MDK reports grants and contracts from the Australian Government National Health and Medical Research Council. AHH reports consulting fees from WHO. All other authors declare no competing interests.
