# Supplementary material for: WHO estimates of the global, regional, and national burden of 42 foodborne infectious and chemical hazards, 2000–21: an updated data synthesis
Source: Lancet Glob Health. 2026 Jun 3;14(8):None. doi: 10.1016/j.langlo.2026.103994 (PMC13433341; doi:10.1016/j.langlo.2026.103994)
Supplement: Supplementary file 1 [file mmc1.pdf]

THE LANCET
Global Health

Supplementary appendix

This appendix formed part of the original submission and has been peer reviewed. We post it as supplied by the authors.

Supplement to: Lake RJ, Devleesschauwer B, Majowicz SE, et al. WHO estimates of the global, regional, and national burden of 42 foodborne infectious and chemical hazards, 2000–21: an updated data synthesis. *Lancet Glob Health* 2026; published online June 3. https://doi.org/10.1016/S2214-109X(26)00156-7.

Supplementary Material for "WHO Estimates of the Global, Regional, and National Burden of 42 Foodborne Infectious and Chemical Hazards, 2000–2021"

Contents

Table S1. Hazards and health states included in WHO Estimates of the Global Burden of Foodborne Diseases 2026 Edition.................2

Table S2. Subregional Clusters for WHO Estimates of the Global Burden of Foodborne Diseases 2026 Edition...................................4

Figure S1. Methodological Framework for World Health Organization Estimates of the Global Burden of Foodborne Diseases............5

Figure S2. WHO Estimates of the Global Burden of Foodborne Diseases 2026 Edition Average Annual Foodborne Disability-Adjusted Life-Years (DALYs) per 100,000 population, by Subregion (see Table S2), 2021...6

Figure S3. Average Annual Years Lived with Disability per 100,000 population due to 42 Foodborne Hazards, by World Health Organization region*, 2000-2021...7

Figure S4. Average Annual Years of Life Lost per 100,000 population due to 42 Foodborne Hazards, by World Health Organization region*, 2000-2021...9

Figure S5. Average Annual Disability-Adjusted Life-Years (DALYs) per 100,000 population due to 42 Foodborne Hazards, by World Health Organization region*, 2000-2021...11

Text S1: Data cleaning...13

Table S3. Estimated Productivity Loss, 2021...14

Text S2. Estimates of Productivity Losses Associated with Global Foodborne Disease, 2021..........................14

Figure S6. Regional Distribution of Estimated Productivity Loss, 2021..15

Figure S7. Nominal productivity loss as percentage of GDP by country per capita income (Spearman's correlation coefficient -0.465) . 17

Figure S8. Variation in productivity loss in lower Middle-Income Countries, 2021...............................18

Table S1. Hazards and health states included in WHO Estimates of the Global Burden of Foodborne Diseases 2026 Edition

**DIARRHOEAL DISEASE HAZARDS**		**HEALTH STATES**
**Viruses**		
1	Norovirus	Diarrhoeal disease
		Vomiting
2	Rotavirus	Diarrhoeal disease
**Bacteria**		
3	*Campylobacter jejuni/coli**	Diarrhoeal disease
		Guillain-Barré syndrome
4	Enteroaggregative *E. coli*	Diarrhoeal disease
5	Enteropathogenic *E. coli*	Diarrhoeal disease
6	Enterotoxigenic *E. coli*	Diarrhoeal disease
7	Shiga toxin-producing *E. coli*	Diarrhoeal disease
		Haemolytic uraemic syndrome
		End-stage renal disease
8	Non-typhoidal *Salmonella enterica*	Diarrhoeal disease
		Invasive salmonellosis
9	*Shigella* spp.	Diarrhoeal disease
10	*Vibrio cholerae*	Diarrhoeal disease
**Protozoa**		
11	*Cryptosporidium* spp.	Diarrhoeal disease
12	*Cyclospora cayetanensis*	Diarrhoeal disease
13	*Entamoeba histolytica*	Diarrhoeal disease
14	*Giardia duodenalis*	Diarrhoeal disease
**INVASIVE DISEASE HAZARDS**		
**Viruses**		
15	Hepatitis A virus	Acute hepatitis
**Bacteria**		
16	*Brucella* spp.	Acute brucellosis
		Chronic brucellosis
17	*Listeria monocytogenes,* perinatal and acquired	Septicaemia
		Central nervous system infection
		Neurological sequelae
18	*Mycobacterium bovis, caprae,* and *orygis*	Tuberculosis
19	*Salmonella enterica* serotypes Paratyphi A, Paratyphi B, and Paratyphi C	Paratyphoid fever
		Intestinal perforation
20	*Salmonella enterica* serotype Typhi	Typhoid fever
		Intestinal perforation
		Gastrointestinal bleed
**Parasites**		
21	*Toxoplasma gondii,* congenital and acquired	Intracranial calcification
		Hydrocephalus
		Central nervous system abnormalities
		Chorioretinitis

		Acute illness
		Post-acute illness
22	*Trypanosoma cruzi*	Chagas disease
23	*Echinococcus granulosus*	Pulmonary cystic echinococcosis
		Hepatic cystic echinococcosis
		Central nervous system cystic echinococcosis
24	*Echinococcus multilocularis*	Alveolar echinococcosis
25	*Taenia solium*	Epilepsy: seizure-free, treated
		Epilepsy: less severe
		Epilepsy: severe
26	*Ascaris* spp.	Ascariasis infestation
		Mild abdominopelvic problems due to ascariasis
		Severe wasting
27	*Trichinella* spp.	Acute trichinellosis
28	*Clonorchis sinensis*	Abdominopelvic problems
29	*Fasciola* spp. and *Fasciolopsis buski*	Abdominopelvic problems
30	Minute intestinal flukes**	Abdominopelvic problems
31	*Opisthorchis* spp. *(Opisthorchis viverrini* and *Opisthorchis felineus)*	Abdominopelvic problems
32	*Paragonimus* spp.	Paragonimiasus
**ENTERIC INTOXICATIONS**		
33	*Clostridium botulinum*	Mild/moderate botulism
		Severe botulism
**CHEMICAL HAZARDS**		
**Organic pollutants**		
34	Dioxin	Male infertility
**Toxins and allergens**		
35	Aflatoxin B1	Hepatocellular carcinoma
36	Aflatoxin M1	Hepatocellular carcinoma
37	Cassava cyanide	Konzo
38	Peanut allergens	Living with peanut-induced allergy
**Metals**		
39	Inorganic arsenic	Lung cancer
		Skin cancer
		Bladder cancer
		Ischaemic heart disease
40	Cadmium	Chronic kidney disease stage 4 and 5
41	Lead	Intellectual disability
		Cardiovascular diseases
42	Methylmercury	Intellectual disability

* and other thermotolerant species

** Includes *Echinostoma* spp., *Heterophyes* spp., *Metagonimus* spp. and other foodborne intestinal trematode species with the exception of *Fasciolopsis buski*.

Table S2. Subregional Clusters for WHO Estimates of the Global Burden of Foodborne Diseases 2026 Edition

WHO regions	Subregional clusters[a]	Countries
African Region (AFR)	AB	Botswana, Equatorial Guinea, Gabon, Mauritius, Namibia, Seychelles, South Africa
	C	Algeria, Angola, Benin, Cameroon, Cabo Verde, Comoros, Congo, Côte d'Ivoire, Eswatini, Ghana, Guinea, Kenya, Lesotho, Mauritania, Nigeria, São Tomé and Príncipe, Senegal, United Republic of Tanzania, Zambia, Zimbabwe
	D	Burkina Faso, Burundi, Central African Republic, Chad, Democratic Republic of Congo, Eritrea, Ethiopia, Gambia, Guinea-Bissau, Liberia, Madagascar, Malawi, Mali, Mozambique, Niger, Rwanda, Sierra Leone, South Sudan, Togo, Uganda
Region of the Americas (AMR)	A	Antigua and Barbuda, Bahamas, Barbados, Canada, Chile, Guyana, Panama, Saint Kitts and Nevis, Trinidad and Tobago, United States of America, Uruguay
	B	Argentina, Belize, Brazil, Colombia, Costa Rica, Cuba, Dominica, Dominican Republic, Ecuador, El Salvador, Grenada, Guatemala, Jamaica, Mexico, Paraguay, Peru, Saint Lucia, Saint Vincent and the Grenadines, Suriname
	C	Bolivia (Plurinational State of), Haiti, Honduras, Nicaragua, Venezuela (Bolivarian Republic of)
South-East Asia Region (SEAR)	B	Indonesia[b], Maldives, Thailand
	CD	Bangladesh, Bhutan, India, Myanmar, Nepal, Democratic People's Republic of Korea, Sri Lanka, Timor-Leste
European Region (EUR)	A	Andorra, Austria, Belgium, Croatia, Cyprus, Czechia, Denmark, Estonia, Finland, France, Germany, Greece, Hungary, Iceland, Ireland, Israel, Italy, Latvia, Lithuania, Luxembourg, Malta, Monaco, Netherlands (Kingdom of the), Norway, Poland, Portugal, Romania, San Marino, Slovakia, Slovenia, Spain, Sweden, Switzerland, United Kingdom of Great Britain and Northern Ireland
	B	Albania, Armenia, Azerbaijan, Belarus, Bosnia and Herzegovina, Bulgaria, Georgia, Kazakhstan, Montenegro, North Macedonia, Republic of Moldova, Russian Federation, Serbia, Türkiye, Turkmenistan
	C	Kyrgyzstan, Tajikistan, Ukraine, Uzbekistan
Eastern Mediterranean Region (EMR)	A	Bahrain, Kuwait, Oman, Qatar, Saudi Arabia, United Arab Emirates
	BC	Djibouti, Egypt, Iran (Islamic Republic of), Iraq, Jordan, Lebanon, Libya, Morocco, Pakistan, Tunisia
	D	Afghanistan, Somalia, Sudan, Syrian Arab Republic, Yemen
Western Pacific Region (WPR)	A	Australia, Brunei Darussalam, Cook Islands, Japan, Nauru, New Zealand, Niue, Singapore, Republic of Korea
	B	China, Fiji, Malaysia, Marshall Islands, Palau, Tonga, Tuvalu
	C	Cambodia, Kiribati, Lao People's Democratic Republic, Micronesia (Federated States of), Mongolia, Papua New Guinea, Philippines, Samoa, Solomon Islands, Vanuatu, Viet Nam

[a] WHO regions (as of September 2023) were subdivided into 17 subregions based on World Bank income classifications.

[b] In accordance with resolution WHA78.25 (2025), Indonesia was reassigned to the WHO Western Pacific Region as of 27 May 2025. In this instance, data analysis had already been completed with data pertaining to Indonesia included in the South-East Asia regional aggregates. WHO regional comparisons must be done with caution.

Figure S1. Methodological Framework for World Health Organization Estimates of the Global Burden of Foodborne Diseases

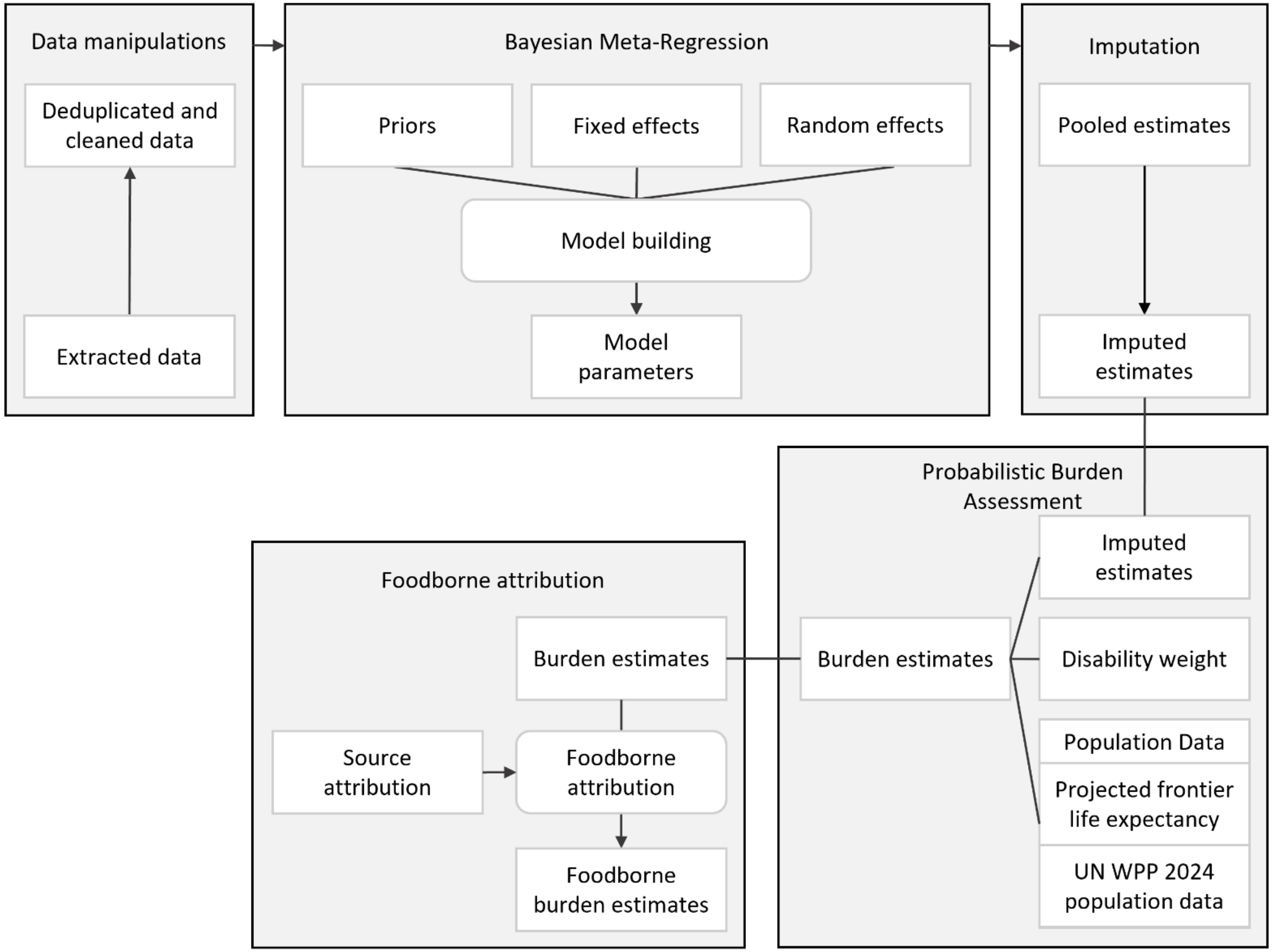

Figure S2. WHO Estimates of the Global Burden of Foodborne Diseases 2026 Edition Average Annual Foodborne Disability-Adjusted Life-Years (DALYs) per 100,000 population, by Subregion (see Table S2), 2021

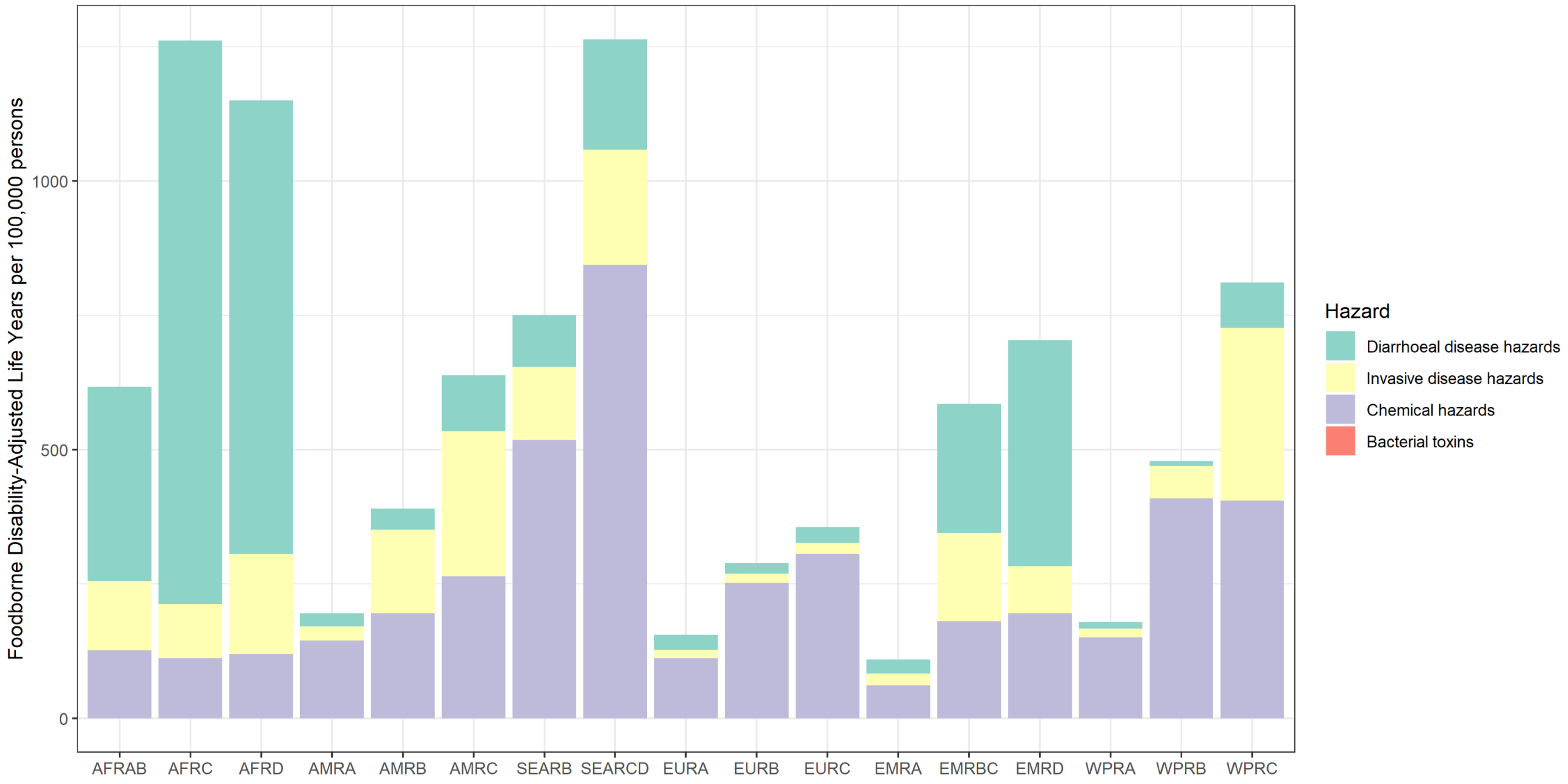

Figure S3. Average Annual Years Lived with Disability per 100,000 population due to 42 Foodborne Hazards, by World Health Organization region*, 2000-2021

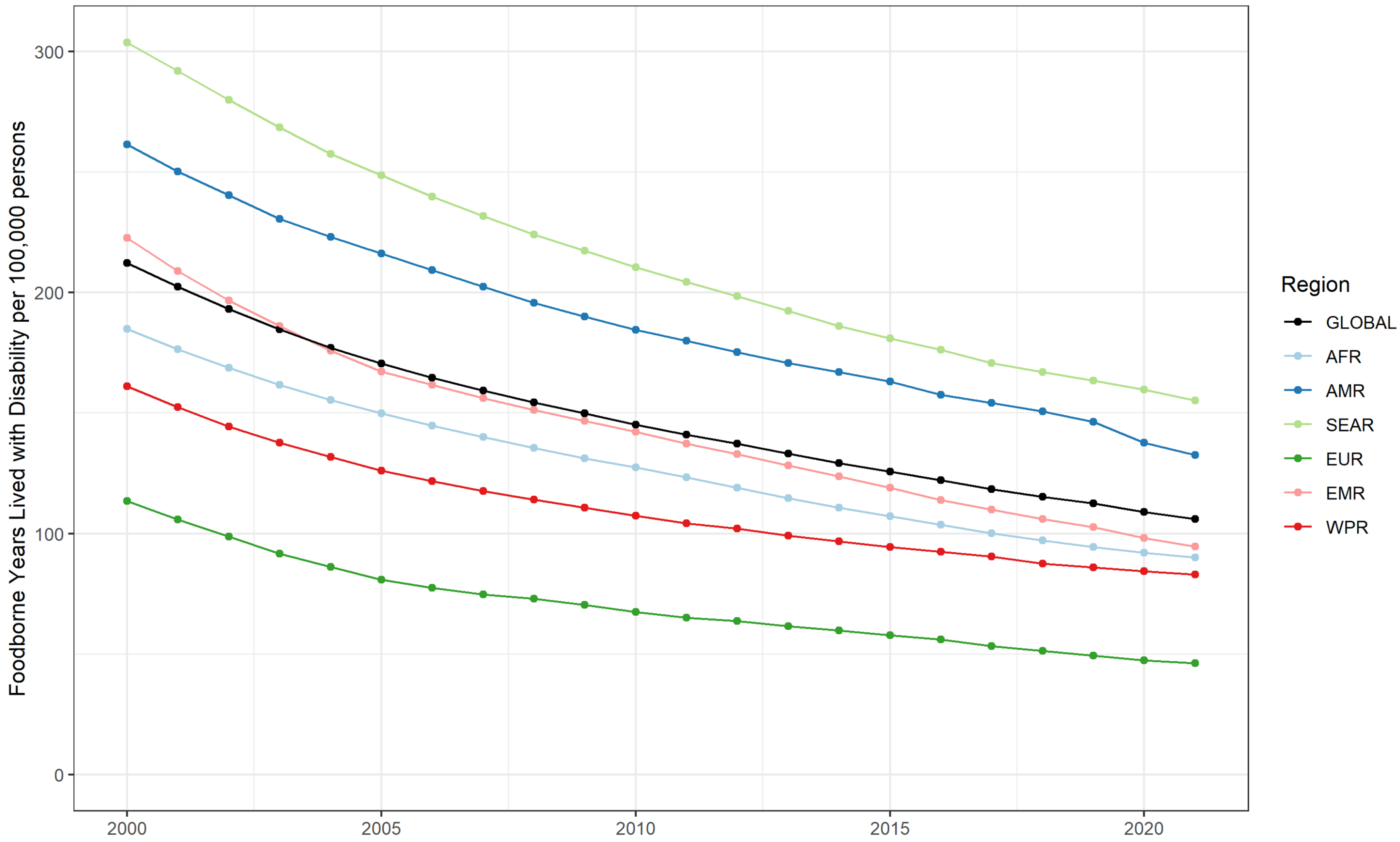

* Countries by WHO Region, as of September 2023. In accordance with resolution WHA78.25 (2025), Indonesia was reassigned to the WHO Western Pacific Region as of 27 May 2025. In this instance, data analysis had already been completed with data pertaining to Indonesia included in the South-East Asia regional aggregates. WHO regional comparisons must be done with caution.:

African Region: Algeria, Angola, Benin, Botswana, Burkina Faso, Burundi, Cabo Verde, Cameroon, Central African Republic, Chad, Comoros, Congo, Côte d'Ivoire, Democratic Republic of the Congo, Equatorial Guinea, Eritrea, Eswatini, Ethiopia, Gabon, Gambia, Ghana, Guinea, Guinea-Bissau, Kenya, Lesotho, Liberia, Madagascar, Malawi, Mali, Mauritania, Mauritius, Mozambique, Namibia, Niger, Nigeria, Rwanda, Sao Tome and Principe, Senegal, Seychelles, Sierra Leone, South Africa, South Sudan, Togo, Uganda, United Republic of Tanzania, Zambia, Zimbabwe

Region of the Americas: Antigua and Barbuda, Argentina, Bahamas, Barbados, Belize, Bolivia (Plurinational State of), Brazil, Canada, Chile, Colombia, Costa Rica, Cuba, Dominica, Dominican Republic, Ecuador, El Salvador, Grenada, Guatemala, Guyana, Haiti, Honduras, Jamaica, Mexico, Nicaragua, Panama, Paraguay, Peru, Saint Kitts and Nevis, Saint Lucia, Saint Vincent and the Grenadines, Suriname, Trinidad and Tobago, United States of America, Uruguay, Venezuela (Bolivarian Republic of)

South-East Asia Region: Bangladesh, Bhutan, Democratic People's Republic of Korea, India, Indonesia, Maldives, Myanmar, Nepal, Sri Lanka, Thailand, Timor-Leste

European Region: Albania, Andorra, Armenia, Austria, Azerbaijan, Belarus, Belgium, Bosnia and Herzegovina, Bulgaria, Croatia, Cyprus, Czechia, Denmark, Estonia, Finland, France, Georgia, Germany, Greece, Hungary, Iceland, Ireland, Israel, Italy, Kazakhstan, Kyrgyzstan, Latvia, Lithuania, Luxembourg, Malta, Monaco, Montenegro, Netherlands (Kingdom of the), North Macedonia, Norway, Poland, Portugal, Republic of Moldova, Romania, Russian Federation, San Marino, Serbia, Slovakia, Slovenia, Spain, Sweden, Switzerland, Tajikistan, Türkiye, Turkmenistan, Ukraine, United Kingdom of Great Britain and Northern Ireland, Uzbekistan

Eastern Mediterranean Region: Afghanistan, Bahrain, Djibouti, Egypt, Iran (Islamic Republic of), Iraq, Jordan, Kuwait, Lebanon, Libya, Morocco, Oman, Pakistan, Qatar, Saudi Arabia, Somalia, Sudan, Syrian Arab Republic, Tunisia, United Arab Emirates, Yemen

Western Pacific Region: Australia, Brunei Darussalam, Cambodia, China, Cook Islands, Fiji, Japan, Kiribati, Lao People's Democratic Republic, Malaysia, Marshall Islands, Micronesia (Federated States of), Mongolia, Nauru, New Zealand, Niue, Palau, Papua New Guinea, Philippines, Republic of Korea, Samoa, Singapore, Solomon Islands, Tonga, Tuvalu, Vanuatu, Viet Nam

Figure S4. Average Annual Years of Life Lost per 100,000 population due to 42 Foodborne Hazards, by World Health Organization region*, 2000-2021

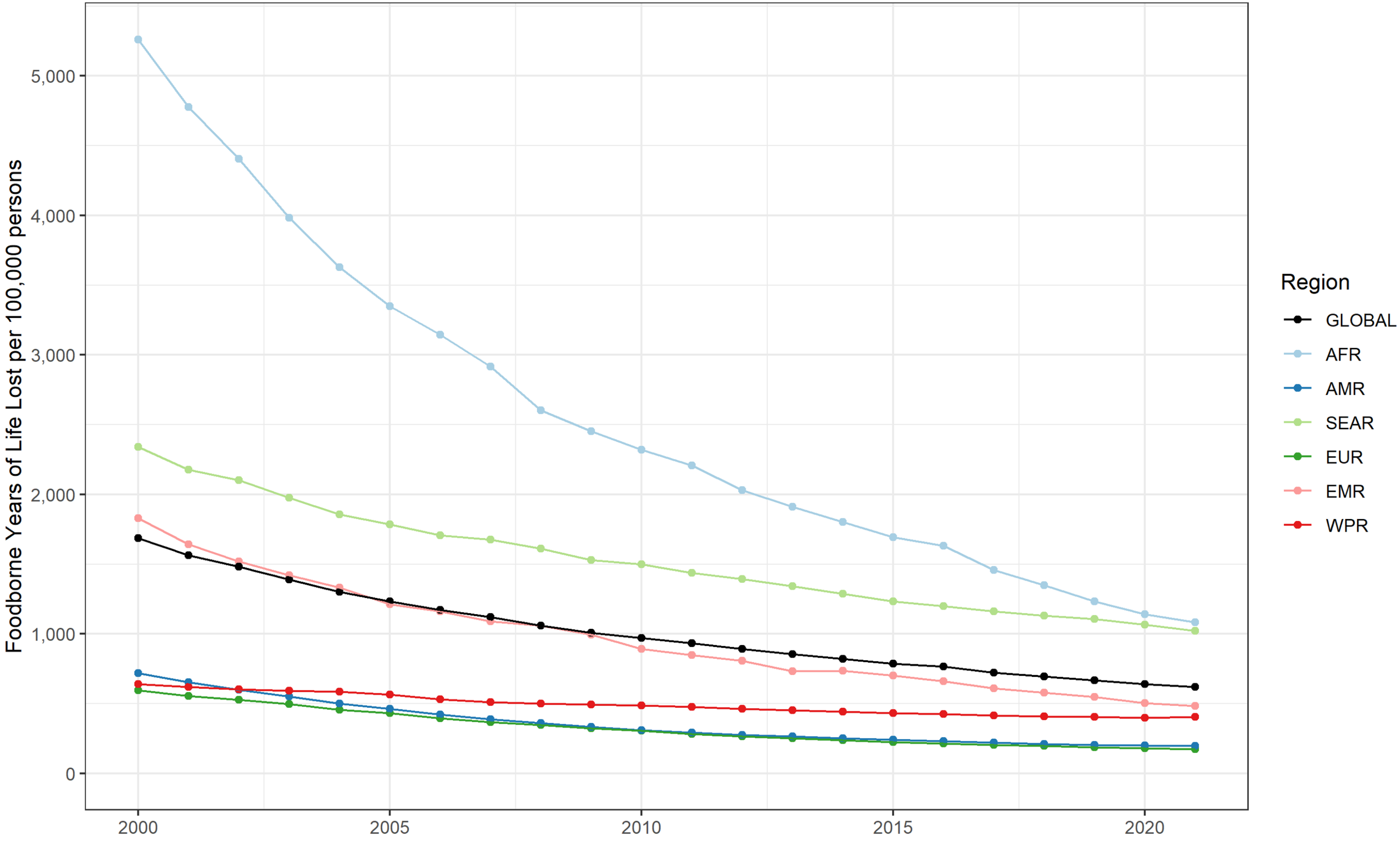

* Countries by WHO Region, as of September 2023. In accordance with resolution WHA78.25 (2025), Indonesia was reassigned to the WHO Western Pacific Region as of 27 May 2025. In this instance, data analysis had already been completed with data pertaining to Indonesia included in the South-East Asia regional aggregates. WHO regional comparisons must be done with caution.:

African Region: Algeria, Angola, Benin, Botswana, Burkina Faso, Burundi, Cabo Verde, Cameroon, Central African Republic, Chad, Comoros, Congo, Côte d'Ivoire, Democratic Republic of the Congo, Equatorial Guinea, Eritrea, Eswatini, Ethiopia, Gabon, Gambia, Ghana, Guinea, Guinea-Bissau, Kenya, Lesotho, Liberia, Madagascar, Malawi, Mali, Mauritania, Mauritius, Mozambique, Namibia, Niger, Nigeria, Rwanda, Sao Tome and Principe, Senegal, Seychelles, Sierra Leone, South Africa, South Sudan, Togo, Uganda, United Republic of Tanzania, Zambia, Zimbabwe

Region of the Americas: Antigua and Barbuda, Argentina, Bahamas, Barbados, Belize, Bolivia (Plurinational State of), Brazil, Canada, Chile, Colombia, Costa Rica, Cuba, Dominica, Dominican Republic, Ecuador, El Salvador, Grenada, Guatemala, Guyana, Haiti, Honduras, Jamaica, Mexico, Nicaragua, Panama, Paraguay, Peru, Saint Kitts and Nevis, Saint Lucia, Saint Vincent and the Grenadines, Suriname, Trinidad and Tobago, United States of America, Uruguay, Venezuela (Bolivarian Republic of)

South-East Asia Region: Bangladesh, Bhutan, Democratic People's Republic of Korea, India, Indonesia, Maldives, Myanmar, Nepal, Sri Lanka, Thailand, Timor-Leste

European Region: Albania, Andorra, Armenia, Austria, Azerbaijan, Belarus, Belgium, Bosnia and Herzegovina, Bulgaria, Croatia, Cyprus, Czechia, Denmark, Estonia, Finland, France, Georgia, Germany, Greece, Hungary, Iceland, Ireland, Israel, Italy, Kazakhstan, Kyrgyzstan, Latvia, Lithuania, Luxembourg, Malta, Monaco, Montenegro, Netherlands (Kingdom of the), North Macedonia, Norway, Poland, Portugal, Republic of Moldova, Romania, Russian Federation, San Marino, Serbia, Slovakia, Slovenia, Spain, Sweden, Switzerland, Tajikistan, Türkiye, Turkmenistan, Ukraine, United Kingdom of Great Britain and Northern Ireland, Uzbekistan

Eastern Mediterranean Region: Afghanistan, Bahrain, Djibouti, Egypt, Iran (Islamic Republic of), Iraq, Jordan, Kuwait, Lebanon, Libya, Morocco, Oman, Pakistan, Qatar, Saudi Arabia, Somalia, Sudan, Syrian Arab Republic, Tunisia, United Arab Emirates, Yemen

Western Pacific Region: Australia, Brunei Darussalam, Cambodia, China, Cook Islands, Fiji, Japan, Kiribati, Lao People's Democratic Republic, Malaysia, Marshall Islands, Micronesia (Federated States of), Mongolia, Nauru, New Zealand, Niue, Palau, Papua New Guinea, Philippines, Republic of Korea, Samoa, Singapore, Solomon Islands, Tonga, Tuvalu, Vanuatu, Viet Nam

Figure S5. Average Annual Disability-Adjusted Life-Years (DALYs) per 100,000 population due to 42 Foodborne Hazards, by World Health Organization region*, 2000-2021

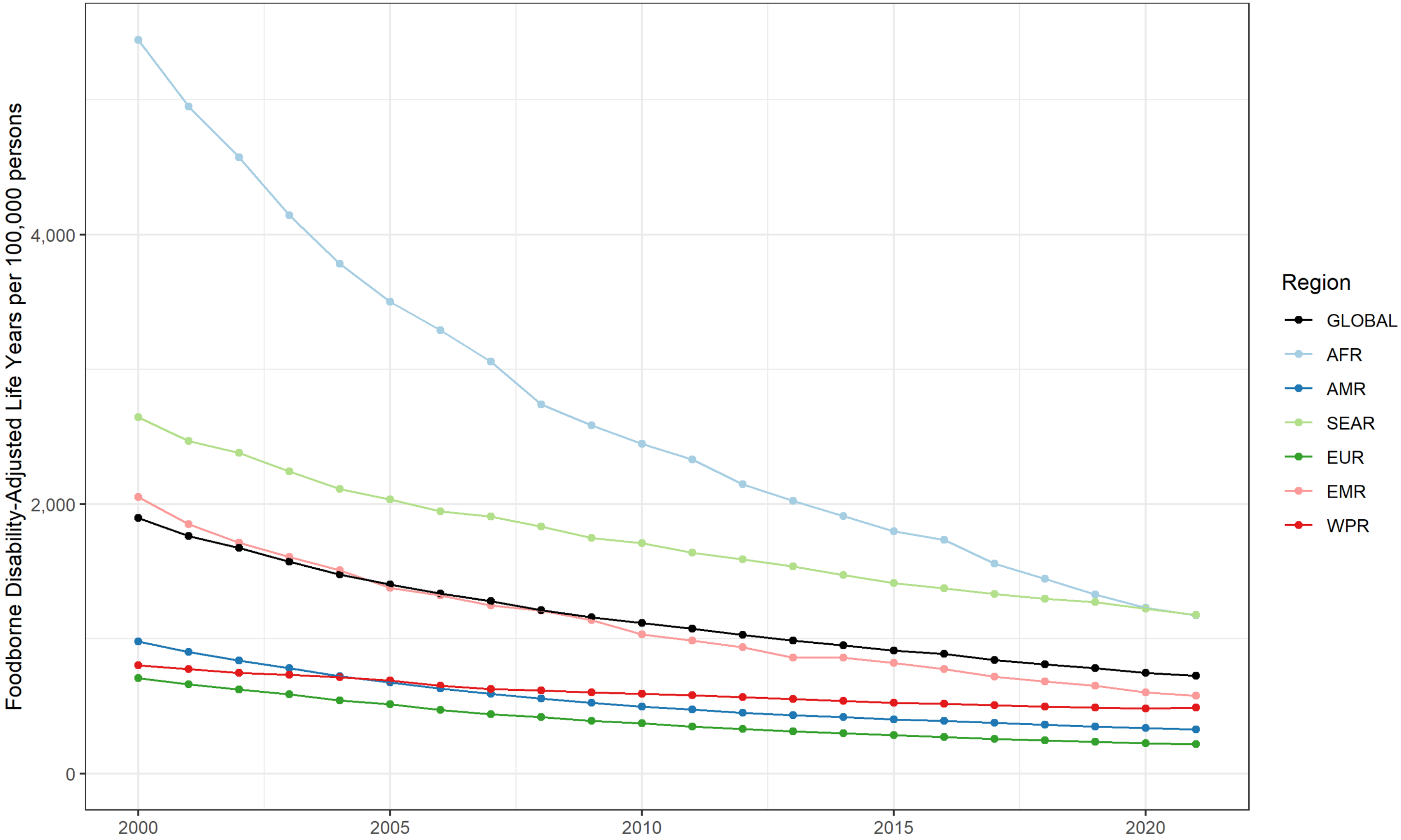

* Countries by WHO Region, as of September 2023. In accordance with resolution WHA78.25 (2025), Indonesia was reassigned to the WHO Western Pacific Region as of 27 May 2025. In this instance, data analysis had already been completed with data pertaining to Indonesia included in the South-East Asia regional aggregates. WHO regional comparisons must be done with caution.:

African Region: Algeria, Angola, Benin, Botswana, Burkina Faso, Burundi, Cabo Verde, Cameroon, Central African Republic, Chad, Comoros, Congo, Côte d'Ivoire, Democratic Republic of the Congo, Equatorial Guinea, Eritrea, Eswatini, Ethiopia, Gabon, Gambia, Ghana, Guinea, Guinea-Bissau, Kenya, Lesotho, Liberia, Madagascar, Malawi, Mali, Mauritania, Mauritius, Mozambique, Namibia, Niger, Nigeria, Rwanda, Sao Tome and Principe, Senegal, Seychelles, Sierra Leone, South Africa, South Sudan, Togo, Uganda, United Republic of Tanzania, Zambia, Zimbabwe

Region of the Americas: Antigua and Barbuda, Argentina, Bahamas, Barbados, Belize, Bolivia (Plurinational State of), Brazil, Canada, Chile, Colombia, Costa Rica, Cuba, Dominica, Dominican Republic, Ecuador, El Salvador, Grenada, Guatemala, Guyana, Haiti, Honduras, Jamaica, Mexico, Nicaragua, Panama, Paraguay, Peru, Saint Kitts and Nevis, Saint Lucia, Saint Vincent and the Grenadines, Suriname, Trinidad and Tobago, United States of America, Uruguay, Venezuela (Bolivarian Republic of)

South-East Asia Region: Bangladesh, Bhutan, Democratic People's Republic of Korea, India, Indonesia, Maldives, Myanmar, Nepal, Sri Lanka, Thailand, Timor-Leste

European Region: Albania, Andorra, Armenia, Austria, Azerbaijan, Belarus, Belgium, Bosnia and Herzegovina, Bulgaria, Croatia, Cyprus, Czechia, Denmark, Estonia, Finland, France, Georgia, Germany, Greece, Hungary, Iceland, Ireland, Israel, Italy, Kazakhstan, Kyrgyzstan, Latvia, Lithuania, Luxembourg, Malta, Monaco, Montenegro, Netherlands (Kingdom of the), North Macedonia, Norway, Poland, Portugal, Republic of Moldova, Romania, Russian Federation, San Marino, Serbia, Slovakia, Slovenia, Spain, Sweden, Switzerland, Tajikistan, Türkiye, Turkmenistan, Ukraine, United Kingdom of Great Britain and Northern Ireland, Uzbekistan

Eastern Mediterranean Region: Afghanistan, Bahrain, Djibouti, Egypt, Iran (Islamic Republic of), Iraq, Jordan, Kuwait, Lebanon, Libya, Morocco, Oman, Pakistan, Qatar, Saudi Arabia, Somalia, Sudan, Syrian Arab Republic, Tunisia, United Arab Emirates, Yemen

Western Pacific Region: Australia, Brunei Darussalam, Cambodia, China, Cook Islands, Fiji, Japan, Kiribati, Lao People's Democratic Republic, Malaysia, Marshall Islands, Micronesia (Federated States of), Mongolia, Nauru, New Zealand, Niue, Palau, Papua New Guinea, Philippines, Republic of Korea, Samoa, Singapore, Solomon Islands, Tonga, Tuvalu, Vanuatu, Viet Nam

Text S1: Data cleaning

General data cleaning was done for each hazard. In a first check incomplete data points were flagged and send back to the reviewers if possible. After review incomplete data points were excluded. Additional exclusions were done for overseas and disputed territories, data before 1990, missing WHO region, missing sample size and missing model parameters. If possible a missing sample size was imputed with the national population. Additional ad hoc decisions (regarding outliers, limited data regions or extensive systematic reviews) were made together with support of the different task forces.

Table S3. Estimated Productivity Loss, 2021

Country Income Group*	Productivity Loss ($ Billion Current)			Productivity Loss as Proportion of National GDP (%)	
	All Ages	Aged 5 Years and Over	Aged Under 5 Years	All Ages	Aged 5 Years and Over
Low Income	5.1	2.4	2.7	1.16	0.52
Lower Middle Income	73.5	52.2	21.3	0.76	0.44
Upper Middle Income	123.6	111.1	12.5	0.38	0.29
High Income	107.5	89.3	18.2	0.20	0.15
All Countries	309.6	255.1	54.6	0.52	0.31

*World Bank Country and Lending Groups, World Bank Classification of Countries by Income Level, (https://datahelpdesk.worldbank.org/knowledgebase/articles/906519-world-bank-country-and-lending-groups) accessed Jan. 8, 2026).

Note: Figures may not sum due to rounding.

Text S2. Estimates of Productivity Losses Associated with Global Foodborne Disease, 2021

Productivity loss is a measure of lost income from labour. This mirrors the approach of the World Bank in its report The Food Safety Imperative (Jaffee et al., 2019). Here this was measured as the product of Gross National Income (GNI) per capita and the national DALY loss by age for each country taken from the global burden estimates. Age breakdowns in the global burden estimates were used to develop separate estimates for those under 5 years of age, 5 years of age and old, all ages. We estimate productivity loss at the national level and then aggregate to regions. GNI per capita is taken from the World Bank's World Development Indicators (World Bank, 2026). The same population estimates are used as for the calculation of the global burden estimates.

For 2021, the estimated productivity loss was US$310 billion in nominal terms for all countries combined when all age groups are considered (Table S3), and US$647 billion at purchasing power parity. High-income countries accounted for approximately $108 billion of this total; the estimated loss among low and middle-income countries was therefore $202 billion. Three countries, namely China, India, and the United States, collectively accounted for nearly 55% of the global total loss of productivity.[1] These three countries are the primary reason why just over three quarters of the global productivity loss is accounted for by three of the WHO regions, namely Western Pacific, South-East Asian, and the Americas (Figure S6).

When analyzing productivity losses, health economists tend to exclude coverage of children, and certainly very young children. Future work will use the WHO 2026 edition findings to estimate annual productivity losses among adult (or working age) populations only, while parallel analysis will be undertaken to estimate longer term 'human capital losses' associated with the burden of food-borne disease among the zero to 14 year cohort.[2] Here, we simply distinguish between the estimated productivity losses for ages 5 and over versus those for the under-5 population. The former probably represents a better approximation of productivity loss in the national economy. For the 5 and over global population, the estimated productivity loss is $255 billion, of which high-income countries account for $89 billion and low and middle-income countries $166 billion.

Figure S6. Regional Distribution* of Estimated Productivity Loss, 2021

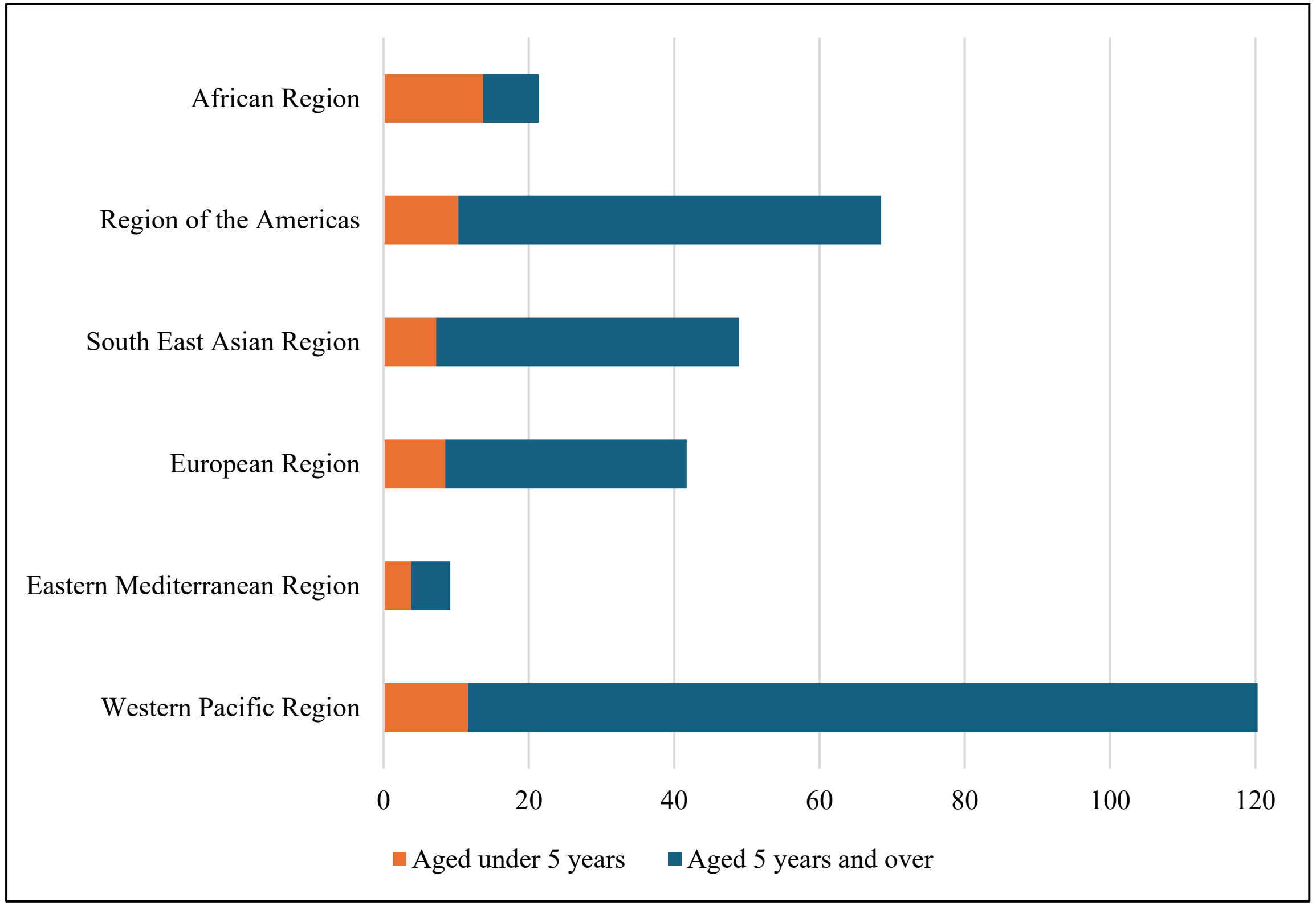

*In accordance with resolution WHA78.25 (2025), Indonesia was reassigned to the WHO Western Pacific Region as of 27 May 2025. In this instance, data analysis had already been completed with data pertaining to Indonesia included in the South-East Asia regional aggregates. WHO regional comparisons must be done with caution.

Productivity loss estimates should be considered in relation to other major economic indicators. Table 1 relates estimated productivity losses to national GDP levels. At the global level productivity losses are equivalent to 0.52% of GDP when all ages are considered and 0.31% for just the losses associated with the 5 and over population. While the monetary estimates of productivity losses are often lower among low-income countries, those losses tend to be higher in proportion to national GDP. Arguably, this may be a better measure of societal losses than simply the numeric estimate.

Country income group and regional aggregates or averages tend to gloss over large variations which occur at the level of individual countries. It is such variations, and the apparent positive or negative outliers which give rise to questions or hypotheses about contributing factors. Figure 2 illustrates the wide variations among countries in terms of the size of the productivity loss relative to national GDP. This ranges from 0.1% to 2.7% globally, yet there are also wide variations among countries at similar levels of per capita income. Similar comparisons can be made in relation to total national food expenditures or other macro or sectoral level indicators.

Figure S7. Nominal productivity loss as percentage of GDP by country per capita income (Spearman's correlation coefficient -0.465)

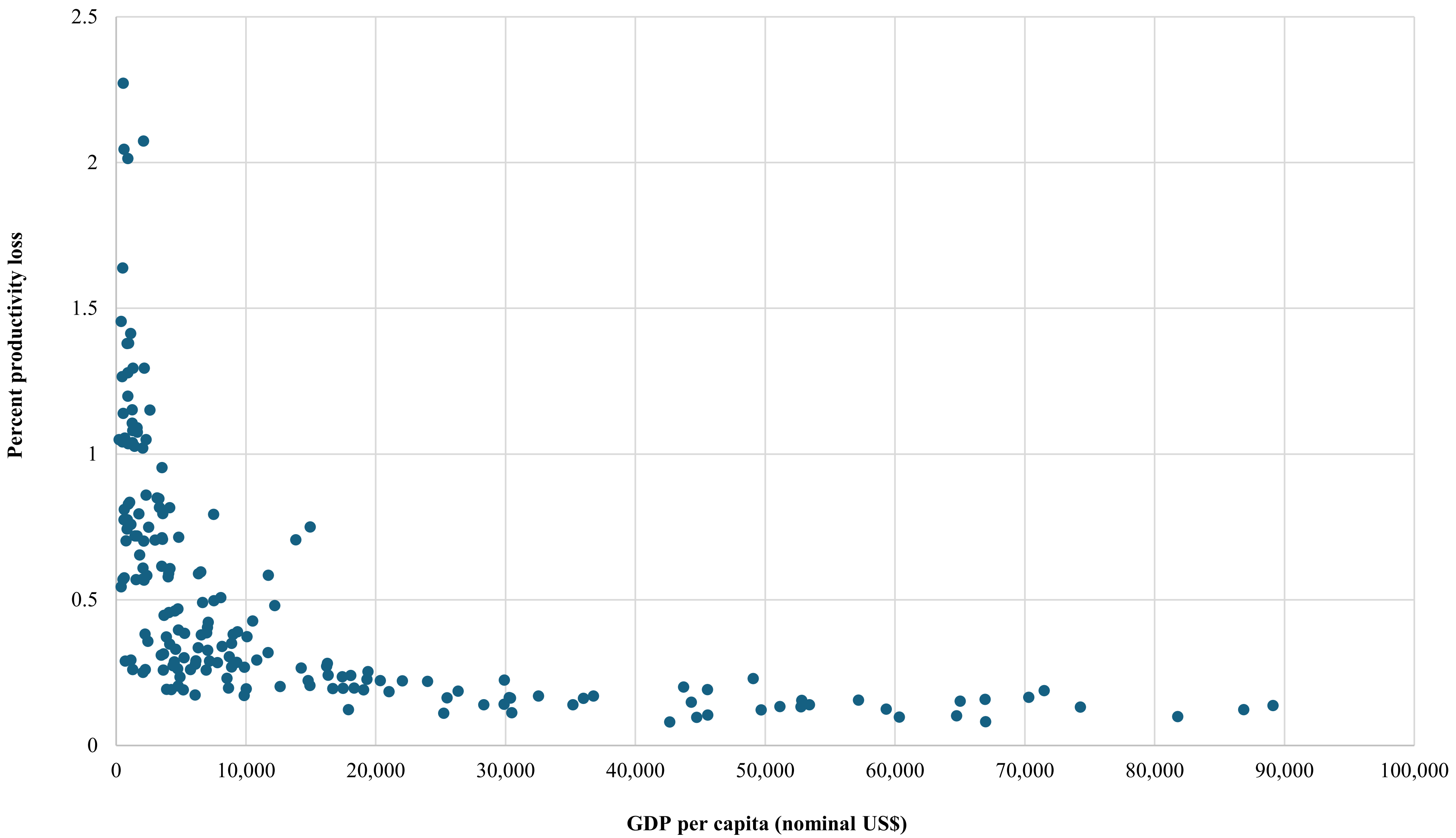

Countries also differ greatly in how the burden of food-borne disease is distributed amongst their population, which translates into the distribution of economic costs or losses by age. See the illustration below (Figure S8) for a select number of lower middle-income countries. Contrast, for example, the situations in India and Nigeria (highlighted). For India, more than 80% of the estimated productivity loss is among the over-5 population; the situation is the reverse in Nigeria where the burden is concentrated among the very young. The figure shows wide variations among these other lower middle-income countries. In most African countries, a large majority of the burden occurs among the very young, such that more emphasis should be placed on assessing longer-term human capital loss (among children) rather than on annual productivity losses among adults.

Figure S8. Variation in productivity loss in lower Middle-Income Countries, 2021

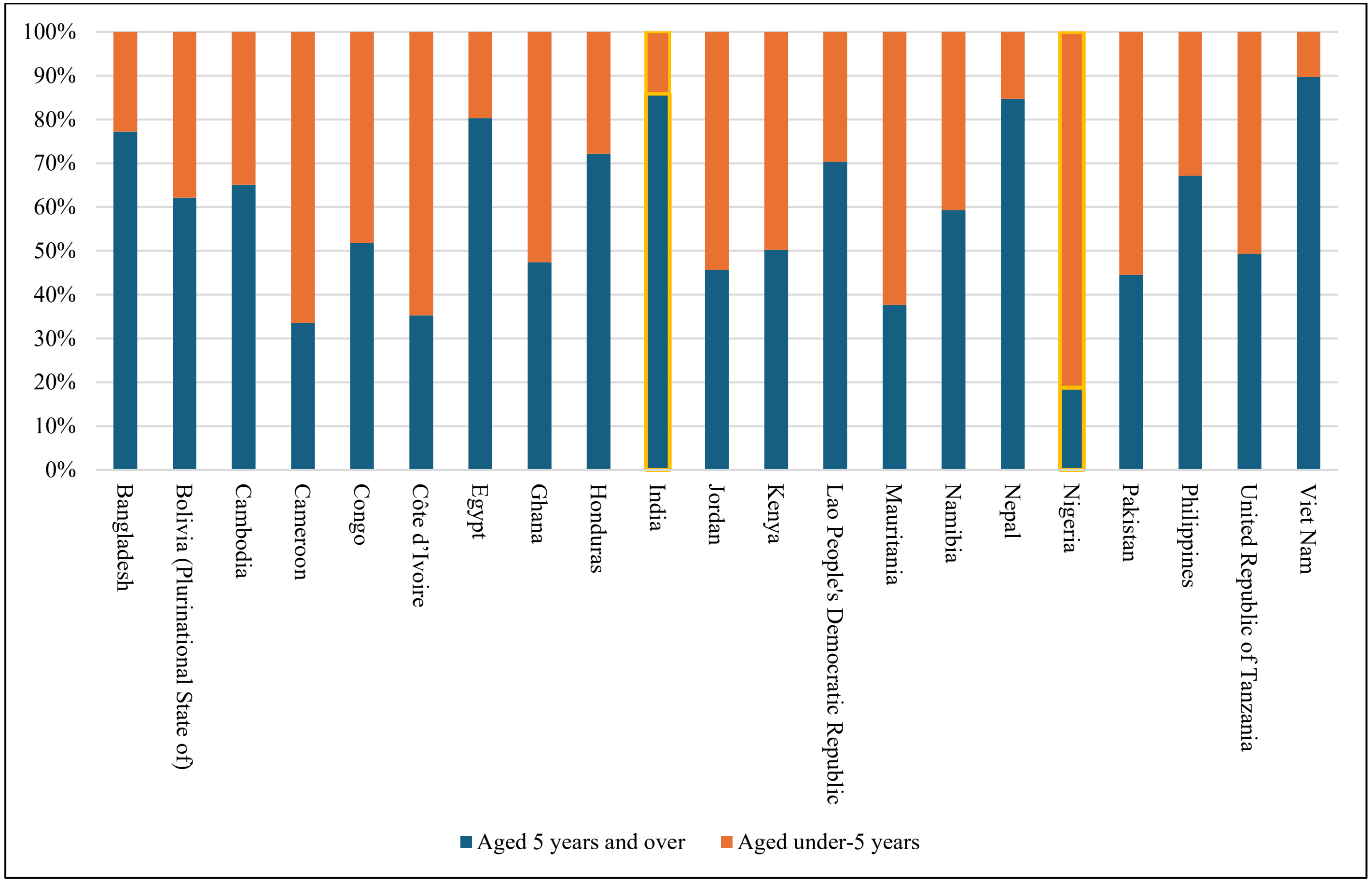

[1] They accounted for 40% of global population in that year.
[2] For some low and lower middle-income countries, children 0-14 constitute forty percent or more of national populations. For such countries, future estimates for 'human capital losses' will likely be far larger than what is estimated here for productivity losses.
[3] While the Under-5 productivity loss calculated represents just 18% of the All-Ages total at the global level, it accounts for half or more of the total productivity loss in 32 countries, including 14 low-income and 14 lower middle-income countries. For such countries, a dedicated analysis of human capital loss is especially important.

Jaffee, S., Henson, S., Unnevehr, L., Grace, D. and Cassou, E. (2019). The Safe Food Imperative: Accelerating Progress in Low- and Middle-Income Countries. World Bank, Washington DC.

World Bank (2026) https://databank.worldbank.org/source/world-development-indicators Accessed 9 Jan 2025
